# Mucosal immune cell populations and the bacteriome of adenoids and tonsils from people living with HIV on suppressive antiretroviral therapy

**DOI:** 10.3389/fmicb.2022.958739

**Published:** 2022-08-11

**Authors:** Olivia Briceño, Mauricio Gónzalez-Navarro, Nadia Montufar, Monserrat Chávez-Torres, Indira Abato, Ariana Espinosa-Sosa, Yuria Ablanedo-Terrazas, Yara Luna-Villalobos, Santiago Ávila-Ríos, Gustavo Reyes-Terán, Sandra Pinto-Cardoso

**Affiliations:** ^1^Centro de Investigación en Enfermedades Infecciosas, Instituto Nacional de Enfermedades Respiratorias “Ismael Cosío Villegas”, Ciudad de México, Mexico; ^2^Comisión Coordinadora de Institutos Nacional de Salud y Hospitales de Alta Especialidad, Secretaría de Salud, Ciudad de México, Mexico

**Keywords:** adenotonsillar microbiota, HIV, tonsillitis, adenoids, oral cavity, oral microbiota, adaptive immunity, innate immunity

## Abstract

Ear, nose, and throat (ENT) conditions are prevalent in people living with HIV (PLWH) and occur at all strata of CD4 counts and despite antiretroviral therapy (ART). ENT conditions are underreported in PLWH. Also, little is known about the adenotonsillar microbiota and its relation to resident adaptive and innate immune cells. To bridge this gap, we characterized immune cell populations and the bacterial microbiota of two anatomical sites (adenoids, tonsils) and the oral cavity. Adenoids and tonsils were obtained from PLWH (*n* = 23) and HIV-seronegative individuals (SN, *n* = 16) after nasal surgery and tonsillectomy and processed for flow cytometry. Nasopharyngeal, oropharyngeal swabs, and oral rinses were collected prior to surgery for 16S sequencing. Wilcoxon rank sum test, principal coordinate analysis, permutational multivariate analysis of variance, and linear discriminant analysis (LEfSe) were used to assess differences between PLWH and SN. Spearman’s correlations were performed to explore interactions between the bacteriome and mucosal immune cells. Of the 39 individuals included, 30 (77%) were men; the median age was 32 years. All PLWH were on ART, with a median CD4 of 723 cells. ENT conditions were classified as inflammatory or obstructive, with no differences observed between PLWH and SN. PLWH had higher frequencies of activated CD4+ and CD8+ T cells, increased T helper (Th)1 and decreased Th2 cells; no differences were observed for B cells and innate immune cells. Alpha diversity was comparable between PLWH and SN at all 3 anatomical sites (adenoids, tonsils, and oral cavity). The impact of HIV infection on the bacterial community structure at each site, as determined by Permutational multivariate analysis of variance, was minor and not significant. Two discriminant genera were identified in adenoids using LEfSe: *Staphylococcus* for PLWH and *Corynebacterium* for SN. No discriminant genera were identified in the oropharynx and oral cavity. Niche-specific differences in microbial diversity and communities were observed. PLWH shared less of a core microbiota than SN. In the oropharynx, correlation analysis revealed that Th17 cells were inversely correlated with bacterial richness and diversity, *Filifactor*, *Actinomyces* and *Treponema*; and positively correlated with *Streptococcus*. Our study contributes toward understanding the role of the adenotonsillar microbiota in the pathophysiology of ENT conditions.

## Introduction

Ear, nose, and throat (ENT) manifestations account for a considerable number of outpatient appointments and are associated with increased morbidity in pediatric and adult populations. ENT pathologies are prevalent in people living with HIV (PLWH) and occur at all strata of CD4 cell counts and despite antiretroviral therapy (ART; Davis et al., 2008; Sanjar et al., 2011; Bao and Shao, 2018). Some of the most common are oropharyngeal (OP) and nasosinusal conditions (Prasad et al., 2006; Sanjar et al., 2011). Despite this, the spectrum of ENT pathologies in HIV infection are underreported, as are their treatment and outcome (Bao and Shao, 2018). Furthermore, some conditions are often refractory to treatment, and ultimately require surgical intervention. Nasal surgery and tonsillectomy are common surgical procedures that aim to alleviate nasal and throat obstruction (Randall, 2020).

Adenoids are located just behind the nose, high up in the throat, while palatine tonsils are at the back of the throat; both are part of the Waldeyer’s ring. Adenoids and tonsils are mucosal inductive sites of humoral and cellular immune responses, and are responsible for mediating homeostasis between the host and the microbiota. Together, they act as gatekeepers by exerting local immune surveillance and by trapping and destroying pathogens that enter through the nose and the mouth (Brandtzaeg, 2011; Randall, 2015; Ritchie et al., 2017). The anatomy, immunological functions and immune cell populations have been previously described (van Kempen et al., 2000; Arambula et al., 2021). Interestingly, histopathological changes in adenoids and tonsils in response to HIV infection have been documented and resemble those described in the other lymph nodes (Wenig et al., 1996).

Recently, the role of the microbiota in respiratory health has been partly elucidated (Man et al., 2017; Esposito and Principi, 2018). The most studied is the oral microbiota (Heron and Elahi, 2017; Annavajhala et al., 2020; Perez Rosero et al., 2021), although information regarding the adenotonsillar microbiota is growing (Johnston and Douglas, 2018), as it may shed light into the pathophysiology of ENT conditions (Brook, 2005; Johnston et al., 2019). Knowledge of the adenotonsillar microbiota is mostly derived from traditional culture-based techniques, although recent studies have used next-generation sequencing (NGS). Collectively, studies suggest that adenoids, and to a lesser extent tonsils, harbor a polymicrobial pathogen reservoir that might colonize adjacent tissues and induce subsequent mucosal inflammation, thus playing an important role in the development of various infectious and non-infectious diseases of the upper airways (Nistico et al., 2011; Johnston et al., 2019). To the best of our knowledge, only one study has addressed the adenotonsillar microbiota in PLWH by NGS (Fukui et al., 2018).

Therefore, we aimed to report the spectrum of ENT manifestations in PLWH attending a national tertiary referral center for HIV care in Mexico City, and comprehensively characterize mucosal immune cell populations and the bacterial microbiota of these anatomical sites (adenoids, tonsils, and the oral cavity). We also explored relationships between mucosal immune cell populations and the nasopharyngeal, oropharyngeal, and oral microbiota.

## Materials and methods

### Ethics statement

Our study was approved by the Ethics Committee and the Ethics in Research Committee of the National Institute of Respiratory Diseases under approval number C04-16, Mexico City, Mexico, and was conducted according to the principles expressed in the Declaration of Helsinki. All subjects were adults (over 18 years old) and provided written informed consent. All subjects agreed to donate their tonsils or adenoid tissue for research. The study was conducted at the Center for Research in Infectious Diseases (CIENI), National Institute of Respiratory Diseases (INER), Mexico City, Mexico.

### Human subjects

Subjects were enrolled between 2016 and 2019 at the National Institute of Respiratory Diseases, Mexico City, Mexico. Our study included both PLWH and HIV-seronegative individuals (SN) who were candidates for tonsillectomy and/or nasal surgery after being examined by the attending ENT surgeon. HIV diagnosis was confirmed by enzyme-linked immunosorbent assay and performed by the Virologic Diagnostic Laboratory, CIENI, INER in accordance with the manufacturer’s instructions (VIDAS HIV DUO Ultra, VIDAS, BioMérieux, Marcy l’Etoile, France and GENSCREEN ULTRA HIV Ag-Ab, Evolis, Bio-Rad, Hercules, CA, United States). Clinical and demographic data was obtained by performing standardized interviews and medical record reviews.

### Surgical intervention

All surgeries were conducted under general anesthesia which consisted of induction with intravenous Fentanyl 3 mg/kg, Propofol at 1–2 mg/kg, and cisatracurium at 100 mg/kg. Orotracheal intubation and anesthesia was maintained with Sevoflurane with a minimum alveolar concentration of 1. Tonsillectomy was performed using standard subcapsular approach with bipolar forceps. Septorhinoplasty using Cottle’s technique and endoscopic sinus surgery were performed according to pathology requirements. Exact surgical techniques are described in detail in Supplementary Material (Annex 1). After preparing and draping the patient, sampling collection was performed within the first 20 mins following anesthesia induction. No local anesthetics or antiseptics were used before the samples were taken (swabs). Standardized postoperative care was followed. Surgical complications were classified using the Clavian-Dindo (C-D) guidelines and the indication to complication index (Dindo et al., 2004; Morand et al., 2021).

### Virologic and immunologic assessments

HIV-1 RNA plasma viral load was determined by automated real-time polymerase chain reaction (PCR) using the m2000 system (Abbot, Abbott Park, IL, United States) with the lower limit of sensitivity of 40 copies/mL. Lymphocyte subsets were obtained by flow cytometry using Trucount in a FACSCanto II (BD Biosciences, San Jose, CA, United States). All clinical testing was performed by the Virologic Diagnostic Laboratory, CIENI, INER.

### Tissue collection and processing

Adenoids and tonsils were surgically removed, immediately placed in R10 media [RPMI-1640 (Lonza, CA, United States) supplemented with 10% fetal bovine serum (FBS, Gibco, MA, United States), 100 U/mL Penicillin/Streptomycin (Gibco, MA, United States), and 1% L-Glutamine (Gibco, MA, United States)] and transported to the laboratory for processing within 30 min. Tissues were cut manually into small pieces and disaggregated by mechanical disruption using a 70 μM nylon mesh filter (BD Biosciences, San José, CA, United States) to obtain a single-cell suspension. Disaggregated cells were washed twice in R10, and red blood cells were lysed using CK (Chloride-Potassium) lysis buffer (Lonza, MD, United States). Single-cell suspensions were manually counted and washed with phosphate-buffered saline (PBS, Lonza, CA, United States) prior to being subjected to flow cytometry-based immunophenotyping.

### Immunophenotyping of mucosal immune cells from adenoids and tonsils

Two separate flow cytometry panels were used; monoclonal antibodies used in each panel are listed in Supplementary Table 1. A total of 5 million cells were stained extracellularly at room temperature during 15 min (panel 1 and panel 2). Cells stained with panel 1 were permeabilized with the eBiosciences FOXP3/fixation permeabilization kit (ThermoFisher Scientific, CA, United States) following the manufacturer’s instructions. Cells were stained intracellularly, incubated for 1 h at room temperature and washed twice with the FOXP3/fixation permeabilization buffer. Finally, cells stained with panel 1 and 2 were washed twice with PBS and were fixed in 300 μl of 1% paraformaldehyde (Sigma-Aldrich, St. Louis, MO, United States) and acquired on a Fortessa LSR cytometer (BD Biosciences). Fluorescence minus one were used as gating controls. Quality controls were performed using BD Cytometer Setup & Tracking Beads (BD Biosciences). A compensation matrix was calculated and applied individually per experiment using BD Comp Beads (BD Biosciences). Data was analyzed using FlowJo V10 software (FlowJo LLC, Ashland, OR, United States). A representative example of the gating strategy used for panel 1 and panel 2 is shown in Supplementary Figures 1, 2. Each population was defined by the expression of surface and intracellular markers as indicated in Supplementary Figures 1, 2.

### Sample collection for microbial 16S sequencing

For subjects undergoing nasal surgery, the nasopharynx and bilateral middle meatus were swabbed; referred to here as the nasopharyngeal (NP) region, with a total of three swabs per subject. For subjects undergoing tonsillectomy, the anterior tonsillar pillar (ATP, right and left), and the tonsillar fossa (TF, right and left), were swabbed, referred to here as the OP region, with a total of four swabs per subject. Additionally, individuals undergoing tonsillectomy were asked to provide an oral rinse (OR), by swirling a sterile saline solution around the mouth. All swabs and OR were taken before surgery. After removal of the palatine tonsils, all subjects were asked to return 6 months after their surgery for a routine examination, and during clinical routine examination, subjects were swabbed again by the same ENT surgeon (follow-up). Upon collection, swabs and oral rinses were placed in a sterile tube, immediately placed on ice, transported to the laboratory and stored at –80^°^C until batch processing. Control swabs were available for both nasal surgeries and tonsillectomies and at baseline and follow-up.

### DNA extraction and 16S rDNA sequencing

Genomic DNA was extracted using the PowerSoil DNA Extraction Kit (MO BIO Laboratories, Carlsbad, CA, United States). For the NP region, each swab was placed consecutively in the same Eppendorf tube supplied by the manufacturer containing the lysis buffer and the lysis enhancer buffer, swirled several times, rinsed against the wall of the tube to remove all liquid and then discarded; so that the three swabs could be extracted and eluted together resulting in a single DNA sample per individual. This was performed to maximize DNA extraction from samples with low biomass. For the OP region, each swab was extracted and eluted separately as we expected sufficient DNA yield from these samples. Each swab was placed in an Eppendorf tube supplied by the manufacturer containing the lysis buffer and the lysis enhancer buffer, swirled several times, rinsed against the wall to remove all liquid and then discarded; resulting in four DNA samples per individual. Oral rinses were centrifuged at 14,000 × *g* for 15 min, the supernatant discarded and the pellet was subjected to DNA extraction. DNA extractions for the NP swabs, OP swabs and OR pellet were performed as instructed by the manufacturer. Briefly, DNA was extracted using both enzymatic and mechanical disruption [the latter by vortexing the tubes horizontally for 30 mins using a vortex-genie 2 (Scientific Industries, Bohemia, NY, United States)]. DNA quantity and quality was evaluated using Nanodrop 1000; extracted DNAS with an A260/A280 ratio above 1.8 were used. 16S libraries were prepared as described in the MiSeq 16S rRNA Gene Amplicon Sequencing protocol (Illumina, San Diego, CA, United States) using the V3–V4 region of the 16S rRNA gene. Primers for this region are the forward (341F): 5′-CCTACGGGNGGCWGCAG-3′; and reverse (805R): 5′- GACTACHVGGGTATCTAATCC-3′ (Klindworth et al., 2013). Primers were bought with the overhang adapter sequences. The forward overhang sequence was: 5′- TCGTCGGCAGCGTCAGATGTGTATAAGAGACAG-followed by the 341F primer; and the reverse overhang sequence was 5′-GTCTCGTGGGCTCGGAGATGTGTATAAG AGACAG-followed by the 805R primer (Invitrogen, Waltham, MA, United States). Libraries were performed as instructed. Briefly, each DNA sample was amplified in triplicate by PCR; three separate PCR reactions in a final volume of 25 μL were performed using the V3–V4 specific primers plus the overhang adapter sequences, 5 ng/μL of DNA, and a Platinum *Taq* DNA High Fidelity Polymerase (Invitrogen). PCR cycling was limited to 25 cycles. PCR amplification was checked on a 2% agarose gel (Sigma-Aldrich, MO, United States). Triplicate PCR reactions were pooled per sample and purified using AgenCourt AMPure XP beads (Beckman Dickson, Atlanta, GA, United States) as instructed by the manufacturer. Dual indices were attached by PCR to the purified amplified PCR amplicon using the Nextera XT Index Kit (Illumina). The index PCR clean-up step was repeated to ensure complete purification of the index libraries using AgenCourt AMPure XP beads. Quantification was performed using the Qubit dsDNA HS Buffer and Standards kit (Invitrogen) as instructed by the manufacturer. Next, each library was normalized to 4 nM by dilution and pooled together. Libraries were checked on a 2100 Bioanalyzer instrument (Agilent Technologies, Santa Clara, CA, United States) and sequenced on the Illumina MiSeq™ platform using a final library concentration of 14 pM to obtain paired-end sequences (2 × 300 cycles). The internal PhiX control was used at 25% (also at 14 pM). Controls were included in parallel to account for reagents contamination during DNA extraction and library preparation. These controls consisted of swabs taken by the ENT surgeon in the operating room and these were processed in the same way. These control swabs did not amplify for the 16S V3–V4 region by 2% agarose gel nor did they yield a quantifiable library, verified on the 2100 Bioanalyzer. 16S library preparation, and sequencing was performed at the CIENI, INER.

### 16S rRNA gene sequence analysis

Raw demultiplexed sequences (without the overhang adapter sequences) were processed with Qiime2 (Quantitative Insights into Microbial Ecology, version 2019.4; Callahan et al., 2016; Bolyen et al., 2019). First, dada2 was used to infer amplicon sequence variants or ASVs in three steps incorporating quality information (filtering), sample inference and removal of chimeras as instructed on the Qiime2 website (Callahan et al., 2016). The Expanded Human Oral Microbiome Database (eHOMD) version 15.2 was trained for the V3–V4 region as instructed on the Qiime2 website. Briefly, the reference dataset (sequences and taxonomy) was obtained from the Silva website, imported into Qiime2, reference reads were extracted using the 341F and 805R primers, and finally, the classifier was trained with the extracted reads and the taxonomy. The classifier was then used to assign taxonomy at 99% similarity (Escapa et al., 2018). Qiime2 artifacts (.qza files, namely the ASV table and the ASV sequences) were imported into R (Phyloseq) for further manipulation, and graph visualization (R V.3.6.2, The R Foundation for Statistical Computing). Diversity analyses were conducted; for alpha diversity two indices were calculated: observed species (hereby termed richness, a qualitative measure of microbial richness) and shannon (a quantitative measure of microbial richness); and for beta diversity, one metric was used: Bray-Curtis dissimilarity (a quantitative measure of community dissimilarity). Permutational multivariate analysis of variance (PERMANOVA) was used to assess differences in microbial community by HIV status or/and anatomical site (default parameters: 999 permutations). Linear discriminant analysis (LDA) effect size (LEfSe) was used to identify differentially abundant taxa between groups and was performed on the Huttenhower Galaxy website (LDA threshold > 4, alpha value for factorial Kruskal–Wallis test 0.05, alpha value for pairwise Wilcoxon test 0.05; Segata et al., 2011). Raw 16S sequences were deposited at the National Center for Biotechnology Information-Sequence Read Archive (NCBI-SRA) under project PRJNA820027.

### Statistical analysis

Differences in demographic and clinical factors between groups were analyzed using the Wilcoxon Rank Sum Test for continuous variables and Fisher‘s exact test for categorical values. Comparisons between two groups were performed using the Wilcoxon Rank Sum Test and between three or more groups using Kruskal–Wallis. For the latter, *p* values were adjusted for multiple comparisons. Two-tailed *p* values less than 0.05 were considered statistically significant. Data was analyzed using the GraphPad Prism 9 software (GraphPad, United States). *p* < 0.05 was considered statistically significant. Correlations were performed using Spearman.

## Results

### Cohort characteristics

Demographic and clinical characteristics stratified by HIV status and by anatomical site are summarized in Table 1, while Supplementary Table 2 summarizes demographic and clinical characteristics stratified by HIV status only. We enrolled 39 individuals, 23 were PLWH, and 16 were SN. The overall median age was 32 years old with no difference observed between PLWH and SN (*p* = 0.73); 9 were female (23%) and all female were SN. PLWH were on ART, had undetectable plasma viral load (<50 copies/mL, except one subject) and presented no evidence of opportunistic infections. Despite ART, the median CD4 T cell count was significantly lower than SN [657 (412–939) vs 1031 (720–1,269), *p* = 0.0046]. The risk factor for HIV infection was unknown. Coinfection with hepatitis C virus was detected only in PLWH (*n* = 3, 13%). Coinfection with hepatitis B virus was detected in 2 (8.69%) PLWH and 1 (6.25%) SN.

**TABLE 1:** Demographic and clinical characteristics stratified by HIV status and anatomical site.

	PLWH (*N* = 23^¥^)			SN (*N* = 16)		
	
	Adenoids	Tonsils	*p*	Adenoids	Tonsils	*p*
**Gender (*n*,%)** M F	12 (100%) 0	12 (100%) 0	>0.999	5 (45.45%) 6 (54.55%)	2 (40%) 3 (60%)	>0.999
Age (mean, min-max)	34 (25–43)	31.42 (19–51)	0.228	33.82 (20–55)	31 (26–34)	0.933
Surgical indications (*n*,%) Inflammatory^1^ Obstructive^2^	2 (16.67%) 10 (83.33%)	11 (91.67%) 1 (8.33%)	<0.001	2 (18.18%) 9 (81.82%)	5 (100%) 0	0.005
PO complications^3^ (*n*,%)	1 (4.17%)	2 (8.33%)	>0.999	1 (6.25%)	0 (100%)	>0.999
HBV (+) serology (*n*,%)	1 (4.17%)	1 (4.17%)	>0.999	0	1 (6.25%)	0.313
HCV (+) serology (*n*,%)	1 (4.17%)	2 (8.33%)	0.590	0	0	>0.999
Smoking (+)^4^ (*n*,%)	4 (33.33%)	6 (50%)	0.680	7 (63.64%)	2 (40%)	0.569
Alcohol consumption (+)^4^ (*n*,%)	7 (58.33)	7 (58.33)	>0.999	8 (72.73%)	5 (100%)	0.509
Use of drugs (+)^4^ (*n*,%)	5 (41.67%)	4 (33.33%)	>0.999	1 (9.09%)	1 (20%)	>0.999
Previous antibiotic therapy^5^ (*n*,%)	3 (25%)	3 (25%)	>0.999	1 (9.09%)	2 (40%)	>0.999
Use of mouthwash^+^ (*n*,%)	7 (45.67%)	8 (66.67%)	>0.999	10 (90.91%)	4 (80%)	>0.999
Use of dental floss^+^ (*n*,%)	5 (33.33%)	6 (50%)	>0.999	7 (63.64%)	2 (40%)	0.597
ART (*n*,%) 2 NRTI + 1 NNRTI 2 NRTI + 1 IP 2 NRTI + 1 INSTI PrEP (TDF/FTC)	9 (75%) 3 (25%) 0 0	10 (83.33%) 0 2 (16.67%) 0	>0.999	–	0 0 0 1 (20%)	–
Presence of oral diseases^x^ (*n*,%) Dental caries Gingivitis Stomatitis	8 (66.67%) 7 (87.5%) 6 (75%) 0 (0%)	9 (75%) 8 (88.89%) 5 (55.56%) 1 (11.11%)	>0.999	8 (72.73%) 8 (100%) 1 (12.5%) 2 (25%)	3 (60%) 3 (100%) 1 (33.33%) 0	>0.999
Time from HIV diagnosis to surgery (months; mean, SD)	74 (7.01)	68.75 (58.91)	0.695	–	–	–
Time since ART initiation to surgery (months; mean, SD)	53.5 (34.47)	53.42 (40.59)	0.893	–	–	–
Time from HIV diagnosis to ART (days; mean, SD)	627.5 (986.8)	501.9 (1,129)	0.439	–	–	–
Plasma viral load (copies/mL; median, IQR) At HIV diagnosis Pre-operative	157,505 (34,703–1,257,650) 40 (40–40)	146,363 (10,993–518,302) 40 (40–44.5)	0.898 0.446	–	–	–
Nadir CD4 (median, IQR)	214.5 (23.75–429.8)	249.5 (67.75–395)	0.525	–	–	–
CD4 cells/mm^3^ (median, IQR)	723 (433.3–956)	592.5 (433.3–916.5)	0.449	941 (635.5–1,254)	1,228 (991.8–1,461)	0.260
CD8 cells/mm^3^ (median, IQR)	958 (630–1,241)	918.5 (773.8–1,207)	0.976	577 (367.5–852.5)	798 (579.5–897.3)	0.414
CD4/CD8 ratio (median, IQR)	0.74 (0.537–1)	0.725 (0.605–0.8975)	0.555	1.58 (1.285–1.78)	1.635 (1.203–2.15)	0.940

Fisher’s exact test and Wilcoxon Rank Sum test were used to compare categorical and continuous variables, respectively.

^¥^Of note, one PLWH has both tonsillectomy and septoplasty performed on the same day, with both adenoids and tonsils been collected for this subject, hence why the number of PLWH does not match with the number of anatomical sites.

^1^Includes chronic and recurrent tonsillitis, tonsil cyst, Thornwaldt cyst and sinusitis.

^2^Includes septal deviation, turbinate hypertrophy and nasal valve collapse. OSAS in case of tonsil’s surgery.

^3^Parosmia, dysgeusia, post-tonsillectomy bleeding and wound infection, respectively.

^4^Formerly or current.

^5^Used formerly or 1 month before the surgery.

^+^Used 2 times per week or more.

^x^Diagnosed at the time of the study.

%, percentage; ART, antiretroviral therapy; HBV, hepatitis B virus; HCV, hepatitis C virus; HIV, human immunodeficiency virus; PLWH, people living with HIV; PrEP, pre-exposure prophylaxis; OSAS, obstructive sleep apnea syndrome; PO, post-operative; mL, milliliter; mm^3^, cubic millimeter; n, number; SD, standard deviation; and SN: seronegative.

**FIGURE 1:**
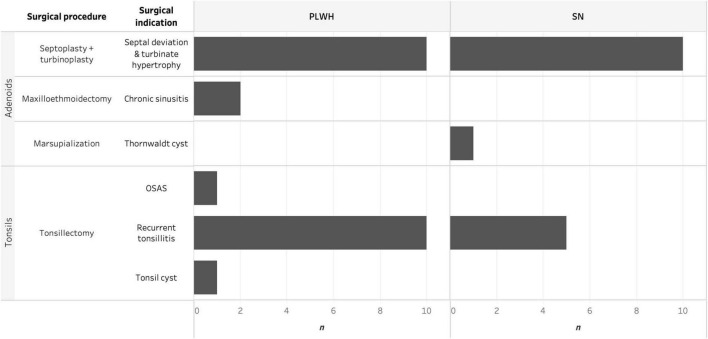
Histogram depicting the surgical procedure and surgical indication for adenoidectomy and tonsillectomy faceted by HIV status and anatomical site. HIV, human immunodeficiency virus; OSAS, obstructive sleep apnea syndrome; PLWH, people living with HIV; and SN, seronegative.

Ear, nose, and throat pathologies and surgical procedures are summarized in Figure 1. Of note, one PLWH had both tonsillectomy and septoplasty plus turbinoplasty performed on the same day, and both adenoids and tonsils were collected from this subject. ENT pathologies were classified into inflammatory or obstructive according to the primary surgical indication; no differences were observed between PLWH and SN (*p* = 0.75). The rate of postoperative complications was 10% and complications were classified as acceptable as described in Methods. One PLWH had a post-tonsillectomy bleeding at day 5 which required a second surgical intervention, while a SN had a wound infection which resolved with oral antibiotics. Two PLWH had temporary sensory dysfunction (dysgeusia for one subject undergoing tonsillectomy and parosmia for one subject undergoing septoplasty); both resolved within a month without any medical intervention. No fatal outcome or sequelae resulted from procedures performed in this study. Prevalence of oral diseases was high, 27 (69.2%) individuals reported having caries, gingivitis, stomatitis or a combination, with no differences observed between PLWH and SN (*p* > 0.99).

### Innate and adaptive cell populations were for the most part similar between adenoids and tonsils

Next, we performed an extensive phenotypic description of innate and adaptive immune cell populations within these two anatomical sites, and included natural killer (NK) cells, macrophages (classical, non-classical, and inflammatory), dendritic cells (myeloid and plasmacytoid), T cells (CD4 and CD8), and B cells. Within CD4 T cells, we further assessed the frequencies of T follicular helper cells (Tfhs), regulatory T cells (Tregs), T helper cells 1 (Th1), Th2, and Th17. Each population was defined by the expression of surface and intracellular markers as indicated in Methods. We also quantified the level of immune activation on total CD4 and CD8 T cells based on the co-expression of CD38 and HLADR. Finally, we assessed the fraction of memory cells (CD45RO+) within total CD4 and CD8 T cells.

**FIGURE 2:**
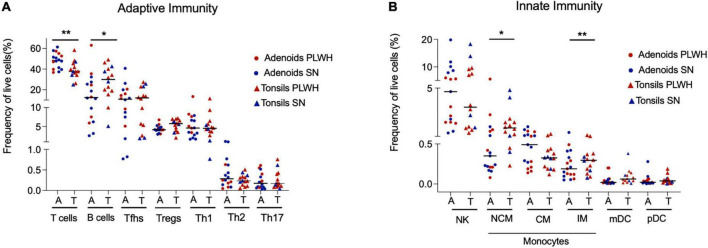
Adenoids and tonsils have similar frequency of adaptive and innate immune cells. Adenoids and tonsils were obtained from PLWH and SN. The frequency (%) of adaptive and innate immunity cells were assessed by flow cytometry. **(A)** The frequencies (%) of T cells (CD3+), B cells (CD19+), and CD4+ T cells subpopulations: Tfhs (CD3+CD4+CD45RO+CXCR5+), Tregs (CD3+CD4+CD45RO+CD25+FOXP3+), Th1 (CD3+CD4+CD45RO+T-bet+), Th2 (CD3+CD4+CD45RO+CRTH2+), and Th17 (CD3+CD4+CD45RO+RORgt+) are shown. **(B)** The frequencies (%) of NK cells (CD56+), macrophages (classical CD14- CD16+, non-classical CD14+ CD16- and inflammatory CD14+ CD16+ HLADR-) and dendritic cells (myeloid dendritic cells CD123- CD11c+ and plasmacytoid dendritic cells CD123+ CD11c-) are shown. Scatter plots were used to present the data. The median is shown by a horizontal line. Each symbol represents one individual. Red triangle: PLWH tonsils, red circle: PLWH adenoids, blue triangle: SN tonsils, and blue circle: SN adenoids. Data was compared using the Wilcoxon Rank Sum test. Only *p* values < 0.05 are shown in the graphs. *p < 0.01; **p < 0.001. %, frequency; CM, classical macrophages; CRTH2, chemoattractant receptor-homologous molecule expressed on TH2 cells; FOXP3, forkhead box P3 protein; HIV, human immunodeficiency virus; IM, inflammatory macrophages; mDC, myeloid dendritic cells; pDC, plasmacytoid dendritic cells; PLWH, people living with HIV; NCM, non-classical macrophages; NK, natural killer; RORgt, retinoic acid-related orphan receptor-gammat; SN, seronegative; Tfhs, T follicular helper cells; Th, T helper; and Tregs, T regulatory.

First, we assessed if there were differences in innate and adaptive immune cell populations by anatomical site (adenoids vs tonsils) in PLWH and SN, and found none (Supplementary Table 3). As the studied cell populations were similar in PLWH and SN, we pooled both tissues to represent the nasopharyngeal-associated lymphoid tissue (NALT) and repeated these analyses comparing adenoids and tonsils, this time irrespective of HIV status. The frequency of adaptive and innate cell populations was similar between adenoids and tonsils (Figure 2), with the exception of non-classical macrophages, inflammatory macrophages and B cells whose frequencies were higher in tonsils compared to adenoids (*p* = 0.011, *p* = 0.0099, and *p* = 0.038, respectively, Figures 2A,B). The frequency of T cells was higher in adenoids compared to tonsils (*p* = 0.0076, Figure 2A). When looking at T cell subpopulations, the frequency of CD4 and CD8 T cells was similar between adenoids and tonsils (Supplementary Figures 3A,B), as were their level of immune activation (Supplementary Figures 3C,D), and their frequency of memory (CD45RO+) CD4 and CD8 T cells (Supplementary Figures 3E,F).

### People living with HIV have higher frequencies of activated CD4+ and CD8+ T cells and increased Th1 cells

**FIGURE 3:**
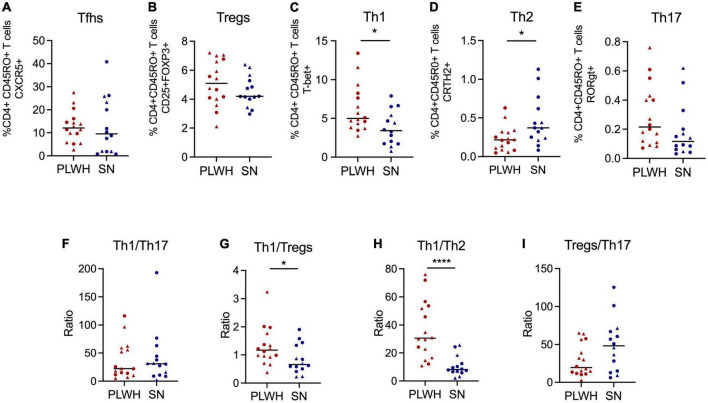
PLWH have increased frequency of Th1 and decreased of Th2 compared to SN. Adenoids and tonsils were obtained from PLWH and SN. Cells from both anatomical sites were pooled to represent the NALT. The frequency (%) of CD4+ T cell subpopulations was assessed by flow cytometry. The frequency of **(A)** Tfhs (CD3+CD4+CD45RO+CXCR5+), **(B)** Tregs (CD3+CD4+CD45RO+CD25+FOXP3), **(C)** Th1 (CD3+CD4+CD45RO+T-bet+), **(D)** Th2 (CD3+CD4+CD45RO+CRTH2+), and **(E)** Th17 (CD3+CD4+CD45RO+RORgt+) are shown. The ratios of **(F)** Th1/Th17, **(G)** Th1/Tregs, **(H)** Th1/Th2, and **(I)** Tregs/Th17 were also determined. Scatter plots were used to present the data. The median is shown by a horizontal line. Each symbol represents one individual. Red triangle: PLWH tonsils, red circle: PLWH adenoids, blue triangle: SN tonsils, and blue circle: SN adenoids. Data was compared using the Wilcoxon Rank Sum test. Only *p* values < 0.05 are shown in graphs. *p < 0.01; **p < 0.001. %, frequency; CRTH2, chemoattractant receptor-homologous molecule expressed on TH2 cells; FOXP3, forkhead box #P3 protein; HIV, human immunodeficiency virus; PLWH, people living with HIV; NALT, nasopharyngeal-associated lymphoid tissue; RORgt, retinoic acid-related orphan receptor-gammat; SN, seronegative; Tfhs, T follicular helper cells; Th, T helper; and Tregs, T regulatory.

Next, we analyzed differences between PLWH and SN. For this analysis, we pooled both anatomical sites (adenoids and tonsils) to represent the NALT. Interestingly, CD4 T cells were significantly reduced while CD8 T cells were significantly increased in PLWH compared to SN (*p* < 0.001, Supplementary Figures 4A,B). Also, CD4 and CD8 T cell activation was significantly increased in PLWH compared to SN (*p* < 0.05, Supplementary Figures 4C,D). Given that our cohort was not well-matched for sex, we looked at differences in immune cell activation by sex within the SN group to assess if these differences confounded our results. We found no differences in immune activation driven by sex in the SN group. The mean CD4 T cell activation was 3.76 [IQR 1.63–8.01] for SN women compared to 5.71 [IQR 3.44–7.7] for SN men (*p* = 0.572), while the mean CD8 T cell activation was 5.20 [IQR 3.05–9.66] for SN women compared to 3.88 [IQR 1.61–5.54] for SN men (*p* = 0.345). No differences were observed for the frequency of memory CD4 and CD8 T cells (Supplementary Figures 4E,F). An increase in Th1 (*p* = 0.024) and a decrease in Th2 (*p* = 0.0188) was observed in PLWH compared to SN (Figures 3C,D). No differences in the frequencies of Tfhs, Th17, and Tregs were observed (Figures 3A,B,E). The ratio Th1/Th17 was similar between PLWH and SN (Figure 3F). Additionally, the ratio Th1/Tregs and Th1/Th2 was significantly increased in PLWH (*p* = 0.034 and *p* < 0.0001, respectively, Figures 3G,H). Of note, the ratio Treg/Th17 was similar between PLWH and SN (Figure 3I). Innate cell populations and B cells were not significantly different between PLWH and SN (Supplementary Figure 5).

### Regional differences in the microbiota of the nasopharynx, oropharyngeal region and oral cavity, with subtle differences between people living with HIV and SN

A total of 69 NP swabs, 99 OP swabs, and 13 OW were available for DNA extraction and 16S sequencing. High quality high number of 16S sequences were only available for nine individuals for the NP region (2 SN and 7 PLWH), 16 individuals for the OP region (11 PLWH and 5 SN), and 10 OW (7 PLWH and 3 SN). Supplementary Tables 4, 5 summarize the demographic, clinical characteristics and oral health/hygiene practices for the NP (*n* = 9) and OP (*n* = 16) cohorts, respectively.

### The genus *Staphylococcus* is enriched in the nasopharyngeal microbiota of people living with HIV

**FIGURE 4:**
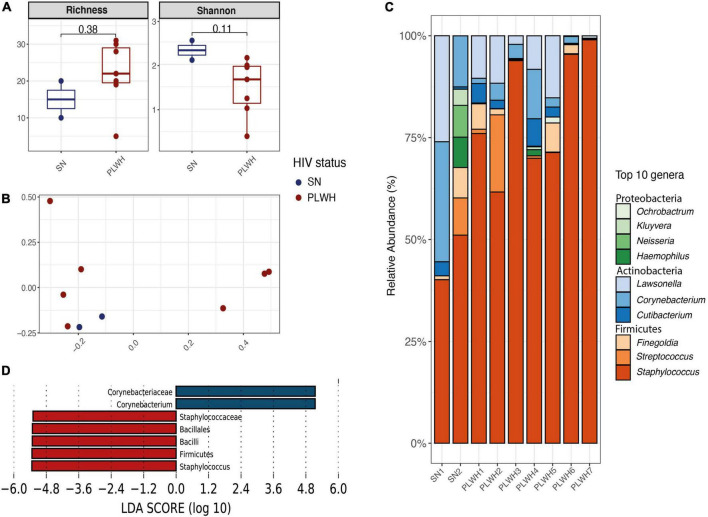
Diversity and bacterial communities of the nasopharynx of PLWH and SN. **(A)** Boxplots showing the median and interquartile range. Two alpha diversity metrics were estimated: richness (number of observed species) and shannon. Groups were compared using the Wilcoxon Rank Sum test. There were no differences between PLWH and SN. **(B)** Principal coordinate analysis of Bray-Curtis dissimilarity, constructed from the ASV table. Samples were clustered according to their HIV status. Samples did not cluster by HIV status (PERMANOVA *p* = 0.407). **(C)** Taxa barplots showing the taxonomic composition at genus level. Only the top 10 genera are represented, these account for 99.5% of all genera present. Genera are listed from the less abundant to the more abundant within each phylum, and ordered in ascending order (Proteobacteria, Actinobacteria, and Firmicutes). Color bars reflect the phyla they belong to and the intensity of the color their relative abundance, with light colors depicting the least abundant, and the darker colors the more abundant. **(D)** Histogram of the LDA scores revealed two differential features taxa by HIV status: *Corynebacterium* was found enriched in SN while *Staphylococcus* enriched in PLWH. LEfSe analyses were performed with LDA values of 4.0 or higher. HIV, human immunodeficiency virus; LDA, linear discriminant analysis; LEfSe, linear discriminant analysis effect size; PCoA, principal coordinate analysis; PERMANOVA, permutational multivariate analysis of variance; PLWH, people living with HIV; and SN, seronegative.

To characterize the bacterial microbiota surrounding enlarged adenoids in the nasopharynx, the middle meatus (bilateral) and nasopharynx were swabbed prior to nasal surgery. A total of 1,145,834 high-quality reads were obtained with a median of 121,006 reads per sample (minimum 10,513–maximum 256,369). As previously mentioned, the cohort was composed of 7 PLWH and 2 SN. All participants were men. Subject information is outlined in Supplementary Table 4 (*n* = 9). Before alpha and beta diversity analysis, samples were rarefied at 9,987 sequences per individual. When stratifying by HIV status, there were no differences in alpha diversity (Figure 4A). We found no clustering by HIV status (beta diversity, *R*^2^ = 0.127, PERMANOVA *p* = 0.407, Figure 4B). The top 5 phyla were *Firmicutes* (79.07%), *Actinobacteria* (17.94%), *Proteobacteria* (2.77%), *Bacteroidetes* (0.21%), and *Fusobacteria* (0.01%; Supplementary Table 6A and Supplementary Figure 6A). An increase in *Firmicutes* and decrease in *Actinobacteria* in PLWH compared to SN was noticeable, though these differences did not reach statistical significance (Supplementary Figure 6B). At genus level, the top 10 genera included *Staphylococcus* (72.8%), *Lawsonella* (8.2%), *Corynebacterium* (7.4%), *Streptococcus* (3.3%), and *Finegoldia* (2.9%; Figure 4C and Supplementary Table 6B). No differences were observed between PLWH and SN (Supplementary Figure 7). Two discriminant genera were identified using LEfSe: *Staphylococcus* for PLWH (LDA = 5.29, *p* = 0.04) and *Corynebacterium* for SN (LDA = 5.33, *p* = 0.04, Figure 4D).

### The oropharyngeal and oral microbiota is similar between people living with HIV and SN

**FIGURE 5:**
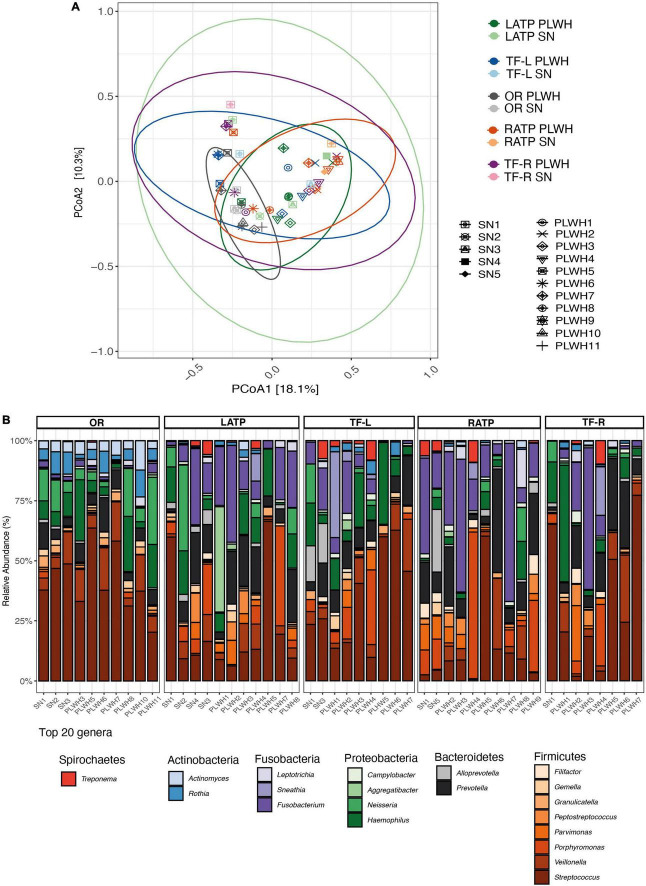
Diversity and bacterial communities of the oropharynx and oral cavity in PLWH and SN. **(A)** Principal coordinate analysis of Bray-Curtis dissimilarity, constructed from the ASV table. Samples were clustered and painted according to their HIV status and the anatomical site. Samples did not cluster by HIV status (PERMANOVA *p* = 0.115). Each symbol represents a subject. Ellipses were added to visualize the bacterial communities at each anatomical site by HIV status. Two ellipses (RATP SN and TF-R SN) are missing given the low number of subjects within each group. **(B)** Taxa barplots showing the taxonomic composition at genus level. Only the top 20 genera are represented, these account for 91.49% of all genera present. Genera are listed from the less abundant to the more abundant within each phylum, and ordered in ascending order (Spirochetes, Actinobacteria, Fusobacteria, Proteobacteria, Bacteroidetes, and Firmicutes). Color bars reflect the phyla they belong to and the intensity of the color their relative abundance, with light colors depicting the least abundant, and the darker colors the more abundant. HIV, human immunodeficiency virus; LATP, left anterior tonsillar pillar; PCoA, principal coordinate analysis; PERMANOVA, permutational multivariate analysis of variance; OR, oral rinse; PLWH, people living with HIV; SN, seronegative; RATP, right anterior tonsillar pillar; TF-L, tonsillar-fossa left; and TF-R, tonsillar fossa-right.

Subjects undergoing tonsillectomy were swabbed at the ATP (right and left) and the TF (right and left), with a total of 4 swabs per subject. Additionally, OR were obtained to assess the overall bacterial composition of the oral cavity. In total, 48 microbial DNA samples were successfully sequenced and used to assess the microbiota of the OP region and the oral cavity (including 11 left ATP (LATP), 9 left TF (TF-L), 10 right ATP (RATP), and 8 right TF (TF-R), and (10 OW). Characteristics of this cohort are summarized in Supplementary Table 5. A total of 7,125,659 high-quality sequences were obtained, with a median of 110,367 sequences per sample. Prior to alpha and beta diversity analysis, rarefaction was performed at 8,462 sequences per sample. No differences in alpha diversity were observed between PLWH and SN (Supplementary Table 7 and Supplementary Figure 8); even when grouping the 4 tonsillar sites to represent the OP region. We found that differences in microbial composition (beta-diversity) were not overall attributable to HIV infection (*R*^2^ = 0.029, PERMANOVA *p* = 0.115). Also, no significant impact of HIV infection was found when analyzing each site separately as determined by PERMANOVA (Figure 5A and Supplementary Table 8). The top 6 phyla were *Firmicutes* (45.5%), *Bacteroidetes* (19%), *Proteobacteria* (15.5%), *Fusobacteria* (14.3%), *Actinobacteria* (4.1%), and *Spirochaetes* (1.25%; Supplementary Table 9A). No differences were observed by HIV status (Supplementary Figure 9). The top 20 genera included *Streptococcus* (26.21%), *Fusobacterium* (11.48%), *Prevotella* (10.16%), *Haemophilus* (8.79%), and *Veillonella* (6.58%; Figure 5B and Supplementary Table 9B). Using LEfSe, no taxa at phylum or genus level were found to discriminate PWLH from SN at each tonsillar site, even when grouping the 4 tonsillar sites to represent the OP region, and at the oral cavity.

### Oropharyngeal microbiota changes after tonsillectomy

Changes in the OP microbiota post-tonsillectomy were also assessed. Four subjects (2 PLWH and 2 SN) returned 6 months after their surgery; the same anatomical regions were swabbed by the same ENT surgeon. In total, 17 microbial DNA samples were successfully sequenced and used to assess the microbiota of the OP region (Supplementary Table 10). The top 6 phyla and top 20 genera and their mean relative abundance at baseline and follow-up are shown in Supplementary Figures 10, 11. Great intra- and interpersonal variability was found, though some patterns seemed to be shared by all four subjects: an increased in *Streptococcus*, and *Veillonella* and a decreased in *Fusobacterium* (Supplementary Figure 12). Also, overall alpha diversity decreased after tonsillectomy, except for one PLWH (Supplementary Figure 13). Both subject (*R*^2^ = 0.37, PERMANOVA *p* = 0.001) and time (*R*^2^ = 0.10, PERMANOVA *p* = 0.009) impacted the OP microbiota (Supplementary Figure 14).

### People living with HIV share less of a core microbiota than SN

Next, we assessed overall differences in the microbiota by anatomical site. After adjusting for multiple comparisons, differences in alpha diversity and beta diversity (*R*^2^ = 0.257, PERMANOVA *p* = 0.001) were found between the nasopharynx, the oropharynx and the oral cavity as expected (Supplementary Figure 15). Next, we calculated the core microbiota (irrespective of HIV status), defined as genera shared by all samples across the three sites (100%) and we found none. When repeating this analysis lowering the threshold to 50%, we found that *Streptococcus* was the only genus shared by all three sites. When repeating this analysis and stratifying by HIV status, we found that PLWH shared less of a core microbiota than SN: the only genus part of the core microbiota of PLWH (50% threshold) was *Streptococcus* while SN shared three genera as part of their core microbiota: *Streptococcus*, *Neisseria*, and *Haemophilus*. A total of 27 discriminant taxa were found using LEfSe (class: anatomical site and subclass: HIV status), with *Corynebacterium* being representative of the nasopharynx, *Fusobacterium*, *Prevotella*, and *Porphyromonas* of the oropharynx, and *Streptococcus, Veillonella*, and *Haemophilus* of the oral cavity (Supplementary Figure 16A). When repeating this analysis considering HIV status, we found that the number of discriminant taxa was higher and distinct in PLWH compared to SN. In SN, *Corynebacterium* and *Staphylococcus* were representative of the nasopharynx, *Alloprevotella*, *Porphyromonas*, and *Prevotellaceace* of the oropharynx, and *Rothia* of the oral cavity (Supplementary Figure 16B). In PLWH, *Lawsonella* and *Staphylococcus* were representative of the nasopharynx, *Fusobacterium*, *Porphyromonas* and *Prevotella* of the oropharynx, and *Neisseria, Haemophilus, Streptococcus* of the oral cavity (Supplementary Figure 16C).

### Relation between immune cell populations and the microbiota

Lastly, we explored relationships between mucosal immune cell populations and the microbiota at each anatomical site. To avoid spurious results and given the small number of individuals, we limited our analyses to genera that were found to discriminate PLWH from SN or genera that we considered of interest and alpha diversity metrics (richness and diversity); the same was done for cell populations, only B cells, CD4 T cell subpopulations and CD4 and CD8 immune activation were considered. For the NP region (adenoids), there was a tendency for Th1 to positively correlate with the shannon index (rho = 0.66, *p* = 0.08) and negatively correlate with *Staphylococcus* (rho = -0.78, *p* = 0.028). Interestingly, Th17 was inversely correlated with CD8 activation (rho = -0.73, *p* = 0.046) and a tendency toward an inverse correlation with *Streptococcus* (rho = -0.71, *p* = 0.057). This tendency for an inverse correlation was also found between Tregs and *Streptococcus* (rho = -0.71, *p* = 0.057). B cells were positively correlated with *Lawsonella* (rho = 0.80, *p* = 0.021) and *Cutibacterium* (rho = 0.81, *p* = 0.022).

For the oral cavity and the OP region, because no discriminating genera were found between PLWH and SN, we explored relationships between the top 20 genera and B cells, CD4 T cell subpopulations and CD4 and CD8 immune activation. No correlations were found in the oral cavity. In the OP region, given that the number of correlations increased substantially, we decided to report only correlation with a rho > 0.50 or <-0.50 and *p* < 0.01. We found that Th17 inversely correlated with richness (rho = -0.70, *p* < 0.001), shannon index (rho = -0.60, *p* < 0.001), *Filifactor* (rho = -0.68, *p* < 0.001), *Actinomyces* (rho = -0.68, *p* < 0.00), and *Treponema* (rho = -0.69, *p* < 0.001); and positively correlated with *Streptococcus* (rho = 0.59, *p* = 0.001). B cells correlated positively with *Filifactor* (rho = 0.67, *p* < 0.001). Tregs correlated positively with *Gemella* (rho = 0.55, *p* = 0.001) while Th2 correlated positively with *Streptococcus* (rho = 0.57, *p* = 0.001) but negatively with *Treponema* (-0.52, *p* = 0.003). No correlations were found between CD4 and CD8 activation, Th1, and the top 20 genera.

## Discussion

Our study provides an insight into ENT conditions in PLWH attending a national tertiary referral center for HIV care in Mexico City. PLWH in this study were on ART with suppressed viremia and presented with CD4 T cell counts above 350 cells/mm^3^. This study was born due to the fact that ENT manifestations are underreported in HIV infection (Barzan et al., 1990). When performing a literature review, we found that few reports had been published in the last 10 years (Bao and Shao, 2018). Also, most of the published literature focused on head and neck malignancies and were mainly described in PLWH with acquired immunodeficiency syndrome (AIDS; Riederer et al., 1990) or case-reports (Sacko et al., 1995). These manifestations were early signs of HIV infection and AIDS (Moazzez and Alvi, 1998), caused by opportunistic and non-opportunistic microorganisms. Very little is known on ENT manifestations in PLWH on ART. We thought to address this gap in the literature; therefore, we present a study drawn from 3 years of experience in a tertiary hospital dedicated to respiratory diseases, where the role of ENT surgeons is critical to both diagnosis and management of these conditions (Barzan et al., 1990). The most common manifestations presented by PLWH on ART were recurrent tonsillitis and septal deviation and turbinate hypertrophy; these ENT conditions were inflammatory or obstructive in nature. The spectrum of ENT conditions was similar to SN and to the general population. Of note, participants with septal deviation and turbinate hypertrophy were slightly older than those with recurrent tonsillitis, this difference in age by anatomical site was observed for PLWH and SN alike. From 2016 to 2019, before the COVID-19 pandemic, 306 patients were admitted to the CIENI, INER; these patients were young, with a median age of 32 years-old, most were men (*n* = 273, 89%) and were scheduled for elective surgery. In total, 39 patients accepted to participate in our research protocol. Elective surgery was performed for a range of ENT conditions. We used a standardized quality assessment for reporting post-operative complication and found no life-threatening, disabling complications and no postoperative sequelae (Dindo et al., 2004; Gonçalves et al., 2020; Morand et al., 2021; Kotisalmi et al., 2022). A recent report concluded that the risk of surgical complications and postoperative mortality in PLWH with controlled HIV and higher CD4 counts was similar to HIV-uninfected individuals (Yan et al., 2021). As part of the preoperative evaluation, the most recent CD4 T cell count and HIV viral load test were reviewed; in our cohort, all PLWH had CD4 T cell counts above 350 cells/mm^3^. The data presented here emphasize that diagnosis and treatment of ENT conditions in PLWH with ART should be considered part of the care management of PLWH to reduce the significant morbidity associated with these conditions and improve their quality of life.

As previously mentioned, most ENT conditions are infectious in nature (Brandtzaeg, 2003). Adenoids and tonsils are part of the NALT; they have a pivotal role in protecting against pathogens that enter the upper respiratory tract (van Kempen et al., 2000; Brandtzaeg, 2011; Randall, 2015). Surgically excised adenoids and tonsils were evaluated by flow cytometry to comprehensively describe the frequency of mucosal innate and adaptive cell populations. Also, to the best of our knowledge, this is the first study to describe CD4+ T cell subpopulations in these two anatomical sites. Pioneer studies reporting the frequencies of immune cell populations, in particular B and T cells, found no glaring differences between these two anatomical sites (Ennas et al., 1984; Hemlin et al., 1993). Another study found a higher frequency of B cells in tonsils (Stanisce et al., 2018). Our results are concordant with the latter, as we also found higher frequency of B cells in tonsils. Indeed, given their location and the type of antigens they encounter, tonsils produce polymeric IgA (and other isotypes) *via* stimulation and differentiation of follicular B lymphocytes into antibody-expressing B memory cells (Brandtzaeg, 1996). In our study, with the exception of a few differences (higher frequency of T cells in adenoids, and inflammatory and non-classical macrophages in tonsils), our results suggest that these two anatomical sites harbor the same innate and adaptive cell populations; this result was unexpected, given their different localization in the upper respiratory tract (Arambula et al., 2021) and the type of antigens they encounter. Tonsils protect against both potentially infectious respiratory pathogens and food antigens; while adenoids are more secluded and protect against inhaled antigens. Interestingly, it is well documented that adenoids and tonsils share similarities in gross and histologic appearance. Histologically, these two anatomical sites consist of well-defined microcompartments that all participate in the immune response. However, tonsils have several unique characteristics. They are lined with stratified squamous non-keratinized or parakeratinized epithelium, while adenoids are lined mainly with a ciliated respiratory epithelium (Arambula et al., 2021). Tonsils are not fully encapsulated, and they do not possess afferent lymphatics. We also found no differences in innate and adaptive cell populations between these two anatomical sites with regards to HIV infection. This is consistent with reports showing that HIV infection causes the same histomorphological changes in adenoids and tonsils; HIV was found to be localized in the follicular dendritic cell network, in multinucleated giant cells, scattered interfollicular lymphoid cells, and cells within the surface or crypt epithelium (Wenig et al., 1996).

Another important facet of our study was to gain a better understanding on the possible changes in cellular composition of adenoids and tonsils in PLWH compared with SN. We found that the frequency of CD4 and CD8 was altered in PLWH. This has previously been reported in other tissues (Eggena et al., 2005). Further examination of CD4 subpopulations provided results of great interest. The frequency of Tfhs, Tregs and Th17 was comparable between PLWH and SN. This was surprising, given that in other lymphoid tissues, such as the gut-associated lymphoid tissue (Gordon et al., 2010; Paiardini and Müller-Trutwin, 2013) or cervical lymph nodes (Paiardini and Müller-Trutwin, 2013), the frequency of these CD4 subpopulations are depleted in PLWH. Interestingly, we found that Th1 cells were significantly increased and Th2 cells significantly reduced in PWLH. We confirmed this pro-inflammatory milieu as the ratio of Th1/Th2 was significantly increased in PLWH. Also, CD4 and CD8 immune activation was higher in PLWH compared to SN. Collectively, these results seem to show a preferential enrichment of pro-inflammatory populations in adenoids and tonsils of PLWH on ART. First, this pro-inflammatory *milieu* may predispose these tissues to fibrosis, atrophy, decreased functional capacity, affecting their function as gatekeepers of the upper respiratory tract (Furler et al., 2019). Also, given that the ENT conditions were similar between PLWH and SN, we argue that these differences might not be attributed to differences in the clinical conditions that required surgical resolution; but rather differences in the antigens that induce the immune response. For example, antigens from microbial exposures. Although it cannot be completely excluded either. Also, given that our cohort was not well-balanced with regards to sex (all women were within the SN group), we cannot exclude that differences in CD4 and CD8 immune activation might be attributable to sex. We explored differences within the SN group by sex comparing CD4 and CD8 immune activation in SN women and SN men, and found no statistical differences. However, we didn’t perform multivariate adjustment due to the lack of statistical power inherent to small cohorts.

The use of NGS has provided great insights into the microbial ecology of human body habitats, including the respiratory tract (Man et al., 2017). In the present study, we used 16S sequencing to describe the NP, OP, and oral microbiota of PLWH with ENT conditions. We found that the NP microbiota was distinct from that of the oral and oropharynx microbiota. Bacterial communities are known to have distinct biogeographic patterns. We also found marked differences in both the richness and diversity of the nasal, oral and OP niche, being significantly lesser in the nasopharynx. As expected, the oral microbiota was the most diverse.

When looking at the NP microbiota, we found that two genera discriminated the NP microbiota of PLWH and SN: *Staphylococcus* for PLWH and *Corynebacterium* for SN. Out of interest, the specie identified was *Staphylococcus capitis*, although we are aware of the difficulty to classify to species level with this approach (16S). We did not find *S. aureus*, but cannot rule out its presence either. Compared to other published studies, species richness was considerably low (Ren et al., 2013). The number of observed bacterial species (richness) varied from 5 to 31, and shannon between 0.4 and 2.6. In our study, the top 3 phyla identified were *Firmicutes*, *Actinobacteria*, and *Proteobacteria*, while in other studies *Firmicutes*, *Proteobacteria*, and *Fusobacteria* were among the predominant (Liu et al., 2011; Ren et al., 2013). In another study, the NP microbiota was dominated by *Proteobacteria*, followed by *Firmicutes*, and *Bacteroidetes* (Bogaert et al., 2011). In our study, we barely detected *Bacteroidetes* and *Fusobacteria*. We found a microbiota characteristic of the skin (Byrd et al., 2018), with *Staphylococcus* and *Cutibacterium*, which thrive in moist areas, like the nasal cavity. Another study found that Staphylococcaceae was present in all nasal samples in an overall relative abundance of 55% (Bassis et al., 2014). We attribute these differences to the type of samples used (adenoid tissue biopsy, adenoid surface swab, and in our case nasopharynx and bilateral meatus media). Our results are similar to those published by Biswas et al. (2015), whom surveyed the nasal microbiota of adult patients with chronic rhinosinusitis, and found a low diverse microbiota dominated by *Actinobacteria* (*Corynebacterium*), *Firmicutes* (*Staphylococcus*), *Proteobacteria* (*Moraxella*) and, in selected cases, *Fusobacteria*, or *Bacteroidetes*. We could not assess the spatial variation as other studies have, because all three swabs were extracted together to yield sufficient material for 16S sequencing. Another important result from Biswas et al. (2015) is that different sides of the nasal cavity contained essentially the same microbiota. It remains to be elucidated whether this is also true when comparing the nasal cavity to adenoids.

Our results suggest that the oral microbiota was not significantly different between PLWH and SN. The oral microbiota has been extensively studied (Li et al., 2022). This is due to the new insights into the role of the latter in periodontal diseases and systemic diseases including cardiovascular diseases (Peng et al., 2022). Results in HIV infection have been inconsistent and contradictory (Li et al., 2021). One study looking at the oral microbiota in ART-treated PLWH found that HIV influenced the oral bacterial communities, albeit this influence was smaller compared to clinical variables like gingivitis, smoking, periodontitis, and antibiotics, which collectively had a stronger significant effect (Griffen et al., 2019). Beck et al. reported that the oral microbiota of PLWH on ART differed significantly from SN and identified *Atopobium* and *Rothia* in the HIV and ART group. Possibly, our cohort was not sufficiently powered to find significant differences between PLWH and SN given the small number of individuals in our cohort, and the prevalence of caries and gingivitis, and smokers. The OP microbiota was also not significantly different between PLWH and SN. To the best of our knowledge, only one study has reported differences in the palatine tonsils in HIV infection (Fukui et al., 2018). Fukui et al. reported that *Firmicutes* was the most abundant phylum in both groups (HIV-infected with and without ART and HIV-uninfected). They also found that *Streptococcus* was significantly increased and *Neisseria*, *Fusobacterium*, and *Haemophilus* were significantly decreased in PLWH. Of note, they also found that the mycobiome did not differ between PLWH and SN. The lack of differences in the mycobiome between PLWH and SN could be due to insufficient sampling to correctly detect fungi (swabs were taken), the primers used and/or insufficient lysis (fungi require more robust DNA extraction; Nash et al., 2017; Auchtung et al., 2018). Nash et al. reported that the internal transcribed spacer 1 (ITS1) had lower amplification efficiency, and incomplete detection of mock communities, compared to ITS2 when both were compared during optimization. ITS1 and ITS2 are commonly used to characterize fungal communities over the 18S rRNA gene, as they provide greater taxonomic resolution. Fungal communities are found at an extremely low abundance (approx. 0.001% to 0.01%). Most fungi found in the gut of healthy adults are from food (and environmental) sources; for example, *Saccharomyces* (bread), *Aspergillus niger* (nuts), *Penicillium* (cheese), *Candida* (diets rich in carbohydrates), and *Malassezia* (skin; Nash et al., 2017; Auchtung et al., 2018). The palatine tonsils could harbor a transient fungal community that reflects predominantly dietary habits, and low-abundance fungi that are disease-associated might be present but not detected; even more so in the absence of overt fungal infection (Auchtung et al., 2018). Fukui’s cohort was composed of 41 HIV-positive (HIV+) participants with CD4 T cell counts above 350 (for the most part), only 6 were severely immunocompromised and 34 HIV+ were on ART. Deeper sequencing depth might be required (Nash et al., 2017). Of course, this hypothesis is driven by results from studies in the gut of healthy adults. Also, oral habits are important (flossing, missing teeth, presence of caries and oral diseases such as gingivitis or periodontitis), and were not reported by Fukui et al., 2018. In a recent study by Fidel and colleagues examining the oral mycobiome and the impact of HIV, ART and clinical variables, they reported that the oral mycobiome clustered into four community types; these community types were defined by the predominance of a single fungi species: *Candida albicans*, *Candida dubliniensis*, *Malassezia restricta*, and *Saccharomyces cerevisiae*. Caries, sampling site (different clinics from which samples came from), gender, HIV/ART, and missing teeth were the variables that most impacted the oral mycobiome in multivariate analyses (Fidel et al., 2021). They also found that HIV parameters like HIV plasma viral load, CD4 cell count, and ART regimen did not affect the oral mycobiome. Also, not reported by Fukui’s was what part of the palatine tonsils was swabbed, that is, were swabs taken from the surface, or the crypts, or both. This could also have impacted their observations as fungi, like bacteria, could reside deep in the crypts. Of interest, the immune status of PLWH did not impact the palatine tonsil (Fukui et al., 2018) or the oral microbiome (Beck et al., 2015). Differences could be attributed to the clinical characteristics of each cohort, and the site (or sites) sampled. Fukui‘s study used swabs, like ours, however, information regarding the exact site of the palatine tonsil swabbed was not available. Interestingly, although no differences were found when analysis each site separately, we did find interesting results when surveying the oropharynx microbiota post-tonsillectomy, and found that microbial diversity decreased; we also found an increase in *Streptococcus*, *Veillonella* and a decrease in *Fusobacterium*. To our knowledge this is the first report to assess differences before and after tonsillectomy, and even if our results should be interpreted with extreme caution due to the limited number of individuals surveyed and the inherent limitation in statistical power, removal of the tonsils might alter the OP microbiota favorably, as suggested by the decrease in *Fusobacterium*, a known pathogen. Further research is needed to corroborate these results. When analyzing differences in the bacterial microbiota of all 3 sites, distinct patterns were found in PLWH and SN, concordant with differences in the core microbiota that characterizes each site. Collectively, these variations in the microbial profile at each anatomical site in both PLWH and SN might be considered as indicative of changes within the upper respiratory tract associated with HIV infection.

A growing body of evidence suggests that changes in key members and/or overall diversity of the microbiome potentially facilitate or impair the body’s natural defense (Pelton, 2012). We explored relationships between mucosal immune cell populations and the resident microbiota. We found that the pro-inflammatory *milieu* was correlated (tendency) with bacterial diversity. Also, Th1 inversely correlated with *Staphylococcus*. Intriguingly, we would expect *Staphylococcus*, a genus of bacteria known to cause severe infections (*S. aureus*) to correlate positively with a pro-inflammatory *milieu*. Because 16S targeted sequencing is not the recommended method for species level identification, we cannot speak to what species are present. These results suggest that increased diversity might trigger a pro-inflammatory response and decrease mucosal clearance in adenoids, leading to hypertrophy and obstruction of the airways. We also found interesting correlations between Th17 and several bacterial in the oropharynx; Th17 were inverse correlated with *Filifactor*, a gram-positive genus of bacteria comprising *F. alocis*, which has recently been identified as a biomarker of periodontal disease (Aja et al., 2021) and in tonsillar cancer patients (De Martin et al., 2021), with *Treponema*, also a genus of bacteria causative of periodontitis (*T. denticola*). Again, these results suggest that bacteria in caries might translocate to and colonize other sites within the oral cavity, and trigger a pro-inflammatory response and decrease mucosal clearance in tonsils, leading to infection (tonsillitis). Interestingly, we found no correlation between CD4 and CD8 activation and genera found at each anatomical site. Given the limitations in our study, we carefully considered reporting these results and interpreting them.

We did not explore correlations between the innate immunity and resident microbial communities nor did we find differences in innate cell population between PLWH and SN. It is important to remember that the first line of defense against pathogens is the innate immune system; acting through their pattern recognition receptors. Unlike the adaptive immune system, the innate immune system is thought to possess no adaptive memory-like responses. However, recent evidence points to the contrary (Sherwood et al., 2022). The role of the microbiota in shaping adaptive immune responses has been extensively reported (Zhao and Elson, 2018). Microbiota-specific adaptive immune responses are generated and maintained by interactions at mucosal epithelial barriers, *via* microbial antigens and/or metabolites (Ansaldo et al., 2021). Innate immune cells also have the capacity to generate memory-like behaviors when exposed to pathogens, eliciting responses to subsequent infection. This memory-like immunity, also termed trained immunity, is thought to be maintained *via* epigenetic modifications (Netea et al., 2016; Sherwood et al., 2022) and respond to microbial antigens and microbiota-derived small extracellular vesicles (Macia et al., 2019). This trained immunity could have deleterious effects for the host, by triggering inflammatory responses to previously encountered microbial antigens. ENT conditions are chronic in nature and pathogenic bacteria associated with these conditions have been shown to produce microbiota-derived small extracellular vesicles (Macia et al., 2019). It is tempting to speculate that innate immune cells in tonsils and adenoids might be primed to recurrent pathogenic microbial antigens, responding more robustly and contributing to inflammation. Whether this response by the innate immune system is different in PLWH and SN remains to be elucidated, and warrants further investigation.

As mentioned previously, nasal surgery and tonsillectomy are common surgical procedures that aim to alleviate nasal and throat obstruction. These surgeries are conducted under general anesthetics, therefore, the impact of anesthetics on immune cell populations and microbial populations is an important factor to consider. Anesthesia has immunosuppressive effects and the breath of its impact is dependent on the type of anesthetic (intravenous or volatile), and the type of surgery and its duration (MedCrave, 2017). The clinical impact of anesthesia on immune cell populations is not currently completely understood. Common anesthetics such as sevoflurane and Propofol have been shown to alter immune function (Martínez-Serrano et al., 2017). Volatile anesthetic agents directly modulate innate immunity by decreasing NK cytotoxicity, and decreasing reactive oxygen species production, chemotaxis, phagocytosis, and cytokine production. On the other hand, they have been shown to cause hypotension and transient hypoxia, which may indirectly promote tissue inflammation and increase cellular adhesion (Stollings et al., 2016; Ackerman et al., 2021). Studies on the clinical impact of anesthesia on the microbiota is limited to the gut microbiota and seem to indicate that volatile anesthetics have an impact on the gut microbiota. In a mouse model, volatile anesthetics (isoflurane) were found to impact the gut microbiota, decreasing its diversity, and depleting commensal bacteria. In this study, impact of volatile anesthetics on the fecal microbiota was assessed 24 h after exposure and compared with before general anesthetic (Serbanescu et al., 2019). Another study also found changes in gut microbial diversity as early as 24 h after mice were given volatile anesthesia (sevoflurane) for 4 h (Han et al., 2021). Given that samples used in this study were taken within the first 20 min following general anesthesia induction, we believe that exposure for such a short time did not affect significantly our results.

We are aware that our study has several limitations. First, complete assessment of the microbiota at each anatomical site was hampered by the number of individuals enrolled and the limited number of samples with high-quality 16S sequencing data. This latter despite checking that the extracted DNA was of sufficient quantity and quality for subsequent analysis. Second, our findings do not allow for mechanisms or causative relationships to be established due to the cross-sectional nature of our study. Large scale longitudinal studies are needed. We obtained samples before and after tonsillectomy, however, COVID-19 pandemic halted our efforts as the INER was converted to a COVID-19 hospital. Further examination of functional immune differences was not performed in our study; but are of great interest to us. We also acknowledge that due to the type of sampling (swabbing), bacteria that reside deep in the crypts, or intracellularly, were unlikely to be detected. Due to the limited number of individuals, we were unable to assess the impact of other variables of interest (clinical variables, the influence of seasonality, and smoking) on the bacterial microbiota at each anatomical site. Given the high prevalence of oral diseases in our cohort, it would have been improved our understanding of the factors that shape the upper respiratory tract microbiota. Finally, our cohort was not well-balanced for sex distribution, as all women were HIV SN. This could have impacted our results. Due to lack of statistical power, we did not adjust for this confounder.

## Conclusion

This work reports the spectrum of ENT manifestations in PLWH attending a national tertiary referral center for HIV care in Mexico City, and comprehensively characterized immune cell populations and the bacterial microbiota of the nasopharynx, oropharynx and oral cavity. Specific patterns of bacterial colonization were found at each anatomical site, with subtle differences accounted for by HIV infection. Relationships between mucosal immune cell populations and the NP and OP microbiota yielded interesting results that suggest pro-inflammatory responses to increased bacterial diversity and exposure to bacterial pathogens. This is one of the first studies to survey both the immune cell populations and the bacterial microbiota of adenoids and tonsils; our results warrant further studies, which are clearly essential to understand the role of the adenotonsillar microbiome in the pathophysiology of ENT conditions.

## Data availability statement

The raw 16S sequences were deposited at the NCBI-SRA under project PRJNA820027.

## Ethics statement

The studies involving human participants were reviewed and approved by Ethics in Research Committees of the National Institute of Respiratory Diseases, Mexico. The patients/participants provided written informed consent to participate in this study.

## Author contributions

SP-C conceived the project, designed experiments, supervised laboratory staff, analyzed and discussed data, and wrote the bulk of the manuscript. NM extracted the DNA from swabs. MC-T extracted DNA from swabs, constructed 16S libraries, and performed 16S rRNA sequencing. MG-N selected the subjects for study, performed surgical procedures, took research samples, collected subject’s data, and contributed some text to Materials and methods, Results, and Discussion. YA-T conceived the project, selected the subjects for study, performed surgical procedures, took research samples, and collected subject’s data. OB conceived the project, designed experiments, performed the flow cytometry experiments, analyzed the flow cytometry data and contributed some text to Materials and methods, Results, and Discussion. AE-S curated databases and analyzed flow cytometry data. YL-V selected the subjects for study and collected subject’s data. IA curated databases and analyzed clinical data. GR-T funding acquisition. SÁ-R data interpretation and editing. All authors read and approved the final manuscript.
