# Supplementary material for: Mucosal immune cell populations and the bacteriome of adenoids and tonsils from people living with HIV on suppressive antiretroviral therapy
Source: Front Microbiol. 2022 Aug 11;13:958739. doi: 10.3389/fmicb.2022.958739 (PMC9404693; doi:10.3389/fmicb.2022.958739)
Supplement: Supplementary file 1 [file Data_Sheet_1.docx]

Supplementary Material

Supplementary Figures and Tables

Annex 1: Detailed information on the surgical techniques used in this study

Supplementary Table 1: Antibodies used for surface and intracellular staining

Supplementary Figure 1: Representative example of the gating strategy for adaptive immune cells

Supplementary Figure 2: Representative example of the gating strategy for innate immune cells and Tfhs

Supplementary Table 2: Demographic and clinical characteristics stratified by HIV status

Supplementary Table 3: Frequency of innate and adaptive immunity is similar in adenoids and tonsils in PLWH and SN

Supplementary Figure 3: The proportion of CD4+ and CD8+ T cells, as well as their levels of activation, is similar in adenoids and tonsils

Supplementary Figure 4: PLWH have decreased frequency of CD4+ T cells, increased frequency of CD8+ T cells and increased levels of CD4+ and CD8+ T cell immune activation

Supplementary Figure 5: PLWH have similar frequency of B cells and innate immune populations compared to SN

Supplementary Table 4: Demographic and clinical characteristics of individuals with nasopharyngeal 16S data (n=9)

Supplementary Table 5: Demographic and clinical characteristics of individuals with tonsillar and oral 16S data (n=16)

Supplementary Table 6: Mean Relative Abundance (%) of the top 5 phyla and 10 genera in the nasopharynx of PLWH and SN

Supplementary Figure 6: Taxa barplots at phylum level, and boxplots comparing the nasopharynx of PLWH with SN

Supplementary Figure 7: Comparison of the top 10 genera of the nasopharyngeal microbiota between PLWH and SN

Supplementary Table 7: Alpha diversity at each anatomical site stratified by HIV status

Supplementary Table 8: R squared and PERMANOVA p for each anatomical site of the oropharynx region and the oral cavity

Supplementary Table 9: Mean Relative Abundance (%) of the top 6 phyla and the top 20 genera in the oropharynx and oral cavity of PLWH and SN

Supplementary Figure 8: Comparison of the mean relative abundance of the top 6 phyla in the oral cavity and oropharynx between PLWH and SN

Supplementary Figure 9: Comparison of the mean relative abundance of the top 6 phyla in the oral cavity and oropharynx between PLWH and SN

Supplementary Table 10: List of reagents, materials, instruments, and software used in this study

**Annex 1: Detailed information on the surgical techniques used in this study**

Tonsillectomy

1. McIvor mouth gag is placed

2. Right tonsil is clamped with Allis forceps for traction

3. An incision through the plica semilunaris and triangularis is made

4. Subcapsular dissection is performed using bipolar forceps from the superior to the inferior pole

5. Packing is placed for hemostasis

6. Same procedure is performed for the left tonsil

7. All packing is removed, if needed further hemostasis is obtained with bipolar forceps

8. Patient is emerged from anesthesia, extubated and taken to recovery ward

Septorhinoplasty

1. With endoscopic visualization 3-4 punch biopsies (with a thru-cut straight Blackesley forceps) are performed in the nasopharynx midline, through the adenoids.

2. Hemostasis is achieved with bipolar forceps

3. 2 ml of 2% lidocaine with adrenaline (1:200000 concentration) in the submucoperichondrial plane at the edge of the septal cartilage

4. An hemitransfixion incision is made

5. Submucoperichondrial flaps are raise bilaterally

6. Anterior, posterior, superior and inferior tunnels are dissected, taking care not to perforate the flap

7. Septal deviation correction is performed accordingly (e.g. basal strip removed for basal deviations). A 1 cm per side L-strut is strictly preserved.

8. Transeptal sutures are placed to hold flaps in place and hemitransfixion is closed, both with 4-0 Polyglactin sutures.

9. Hemostasis is performed as needed

10.  Doyle silastic splints are placed bilaterally

11.  Patient is emerged from anesthesia, extubated and taken to recovery ward

Endoscopic Sinus surgery

1. With endoscopic visualization 3-4 punch biopsies (with a thru-cut straight Blackesley forceps) are performed in the nasopharynx midline, through the adenoids.

2. Hemostasis is achieved with bipolar forceps

3. Middle turbinate is medialized to improve exposure

4. Uncinectomy is performed with back-biting forceps in the inferior aspect, resecting the upper aspect with the debrider

5. Identification of the natural ostium of the maxillary sinus before enlarging the opening with cutting forceps

6. Ethmoid bulla is identified and removed as well as all anterior ethmoid cells, frontal sinus is identified, the lamina papyracea and anterior ethmoidal artery are preserved at all times

7. Basal lamella is identified and penetrated in order to access the posterior ethmoid

8. Skull base is identified and ethmoid cells inferior to it are removed

9. Sphenoid sinus is approached and enlarged transnasally

10.  According to the disease the following were performed: maxillary antrostomy, anterior ethmoidectomy, posterior ethmoidectomy and sphenoidotomy

11.  Hemostasis is performed as needed

12.  Absorbable sponge packing is placed

13.  Patient is emerged from anesthesia, extubated and taken to recovery ward

**Supplementary Table 1: Antibodies used for surface and intracellular staining**

| Marker | Fluorochrome | Clone | Manufacturer |
| --- | --- | --- | --- |
| **Panel 1** | | | |
| CD3 | BV570 | UCHT1 | Biolegend |
| CD4 | APC-Cy7 | A161A1 | Biolegend |
| RORγt | BV421 | Q21-559 | BD |
| CD38 | BV711 | HIT2 | Biolegend |
| HLA-DR | BV785 | L243 | Biolegend |
| CD45RO | BV650 | UCHL1 | Biolegend |
| CRTH2 | FITC | BM16 | Biolegend |
| CD25 | PECy7 | BC96 | Biolegend |
| FOXP3 | PE-CF594 | 206D | Biolegend |
| T-bet | PE | 04-16 | BD |
| CD161 | APC | HP-3G10 | Biolegend |
| live/dead | AmCyan |  | ThermoFisher Scientific |
| CD8 | BV605 | HP-3G10 | Biolegend |
| CD19 | BV510 | HIB19 | Biolegend |
| CD14 | BV510 | M5E2 | Biolegend |
| CD56 | BV510 | HCD56 | Biolegend |
| CD11c | BV510 | 3.9 | Biolegend |
| CD123 | BV510 | 6H6 | Biolegend |
| **Panel 2** | | | |
| CD3 | BV570 | UCHT1 | Biolegend |
| CD4 | PE | A161A1 | Biolegend |
| CD14 | Pacific Blue | M5E2 | Biolegend |
| CD56 | BV711 | HCD56 | Biolegend |
| CXCR5 | PECF-594 | J252D4 | Biolegend |
| HLA-DR | BV785 | L243 | Biolegend |
| CD19 | BV650 | HIB19 | Biolegend |
| PD-1 | BV605 | EH12.2H7 | Biolegend |
| CD16 | APCCy7 | 3G8 | Biolegend |
| live/dead | AmCyan |  | ThermoFisher Scientific |
| CD69 | PECy7 | FN50 | Biolegend |
| CD123 | APC | 9F5 | BD |
| CD11c | PECy5 | B-ly6 | BD |

Abbreviations: APC: Allophycocyanin, BD: Becton Dickinson, BV: Brilliant Violet, CD: cluster of differentiation, CRTH2: chemoattractant receptor-homologous molecule expressed on TH2 cells, FITC: Fluorescein isothiocyanate, FOXP3: forkhead box P3, HLA-DR: human leucocyte antigen-DR, PE: Phytoerythrin, RORγt: retinoic acid-related orphan receptor-gammat

**Supplementary Figure 1: Representative example of the gating strategy for adaptive immune cells**

**Legend:**

Time, singlets and morphology were used to clean the data from unwanted events. Next, dead cells and unwanted populations were excluded (aqua dye positive events and CD14, CD19, CD11c, CD123, CD56, respectively). Live cells were gated on CD3+ and then on CD4+ or CD8+ T-cells. On total CD4+ and CD8+ T cells, the co-expression of CD38 and HLADR (immune activation) and the expression of CD45RO (memory) was performed. On memory CD4+ T cells (CD45RO+) we defined Th1 as T-bet+, Th2 as CRTH2+, Tregs as CD25+FOXP3+ and Th17 as RORγt+ populations.

Abbreviations: CRTH2: chemoattractant receptor-homologous molecule expressed on TH2 cells, FOXP3: forkhead box P3, HLA-DR: human leucocyte antigen-DR, RORγt: retinoic acid-related orphan receptor-gammat, Th: T helper cells

**Supplementary Figure 2: Representative example of the gating strategy for innate immune cells and T follicular helper cells (Tfhs)**

**Legend:** Time, singlets and morphology were used to clean the data from unwanted events. Next, dead cells were excluded (aqua dye positive events). Live cells were gated on CD3+ CD4+ T-cells and Tfhs were identified by the expression of CXCR5. On the CD3-CD4- population, macrophages were defined as: classical (CD3-CD14+CD16-), intermediate (non-classical) (CD14+CD16+) and inflammatory (CD3-CD14-CD16+HLADR-). On the CD14-CD16- fraction, NK cells were gated on the CD56+ population. On the CD3-CD14-CD16-CD56- fraction, B cells were defined as CD19+ population. Plasmacytoid dendritic cells were gated on the CD3-CD14-CD16-CD56-CD19-CD123+CD11c- while myeloid dendritic cells were defined as CD3-CD14-CD16-CD56-CD19-CD123-CD11c+.

Abbreviations: CD: cluster of differentiation, CXCR5: C-X-C chemokine receptor type 5, NK: natural killer cells, Tfhs: T follicular helper cells

**Supplementary Table 2: Demographic and clinical characteristics stratified by HIV status**

|  | PLWH | SN | P value |
| --- | --- | --- | --- |
| N | 23 | 16 |  |
| Gender, n (%)  Female | 0 (0) | 9 (56.25) | **<0.0001** |
| Residence^¥^  Mexico City  Others | 17 (73.91)  6 (26.09) | 9 (56.25)  7 (43.75) | 0.31 |
| Age (Years), median (min-max) | 32 (19-51) | 31.50 (20-55) | 0.73 |
| Smoking^1^, n (%) | 10 (45.45) | 9 (56.25) | 0.74 |
| Alcohol^1^, n (%) | 14 (66.67) | 13 (81.25) | 0.46 |
| Use of mouthwash^2^, n (%)  Yes  Sometimes/No | 11 (52.38)  10 (47.62) | 5 (31.25)  11 (68.75) | 0.19 |
| Use of dental floss^2^, n (%)  Yes  Sometimes/No | 7 (33.33)  14 (66.67) | 3 (18.75)  13 (81.25) | 0.46 |
| Oral diseases^3^  Yes | 16 (84.21) | 11 (78.57 | >0.99 |
| CD4 T cell count^4^, median (IQR) | 657 (412-939) | 1031 (720-1269) | **0.0046** |
| Tissue collected  Adenoids  Tonsils | 12  12 | 11  6 | NA |
| Surgical Indications^5^, n (%)  Inflammatory  Obstructive | 12 (50)  12 (50) | 7 (43.75)  9 (56.25) | 0.75 |
| PO complications^6^, n (%) | 3 (13.04) | 1 (6.25) | 0.63 |

Differences in demographic and clinical factors between groups were analyzed using the Wilcoxon Rank Sum Test for continuous variables and Fisher`s exact test for categorical values.

^¥^Others include the State of Mexico and Morelos

^1^Former or current smokers, data was missing for 1 PLWH for smoking and 2 PLWH for alcohol consumption

^2^Data was missing for 2 PLWH

^3^Data was missing for 4 PLWH and 2 SN. Oral diseases include caries, gingivitis, stomatitis, caries and gingivitis.

^4^Data was missing for 3 SN

^5^Classified according to the primary surgical indication. Chronic and recurrent tonsillitis, tonsil cyst, Thornwaldt cyst and sinusitis were classified as inflammatory conditions, while septal deviation, turbinate hypertrophy, and obstructive sleep apnea syndrome were considered obstructive conditions. One PLWH had both tonsillectomy and septoplasty plus turbinoplasty performed on the same day, and both adenoids and tonsils were collected from this subject.

^6^For PLWH: Parosmia, dysgeusia and post-tonsillectomy bleeding; for SN: wound infection

Abbreviations: %: percentage, HIV: human immunodeficiency virus, IQR: interquartile range, PLWH: people living with HIV, PO: post-operative, max: maximum, min: minimum, n: number, NA: not applicable, SN: seronegative

**Supplementary Table 3: Frequency of innate and adaptive immune cells is similar in adenoids and tonsils of PLWH and of SN**

| **Adaptive Immunity PWLH** | | | | | | | | | | | | | | |
| --- | --- | --- | --- | --- | --- | --- | --- | --- | --- | --- | --- | --- | --- | --- |
|  | T cells | | B cells | | Tfhs | | Tregs | | Th1 | | Th2 | | Th17 | |
|  | Adenoids | Tonsils | Adenoids | Tonsils | Adenoids | Tonsils | Adenoids | Tonsils | Adenoids | Tonsils | Adenoids | Tonsils | Adenoids | Tonsils |
| Median | 96 | 95.55 | 12.9 | 27.35 | 11.4 | 12.05 | 4.41 | 5.68 | 6.37 | 4.78 | 0.19 | 0.22 | 0.31 | 0.17 |
| IQR | 93.05 - 96.83 | 94.7 - 96.83 | 5.94-  35.5 | 18.2-71.75 | 8.83-16.10 | 5.67-16.50 | 3.84 - 5.87 | 3.9 - 7.01 | 4.3-  9.53 | 3.79-7.23 | 0.07-0.34 | 0.1-0.32 | 0.16-0.56 | 0.10-0.40 |
| **P value** | *0.8133* | | *0.2698* | | *0.9819* | | *0.4278* | | *0.4923* | | *0.6354* | | *0.4438* | |
| **Adaptive Immunity SN** | | | | | | | | | | | | | | |
|  | T cells | | B cells | | Tfhs | | T regs | | Th1 | | Th2 | | Th17 | |
|  | Adenoids | Tonsils | Adenoids | Tonsils | Adenoids | Tonsils | Adenoids | Tonsils | Adenoids | Tonsils | Adenoids | Tonsils | Adenoids | Tonsils |
| Median | 94.35 | 82.05 | 11.7 | 32.9 | 9.52 | 12.69 | 4.17 | 5.93 | 3.4 | 2.89 | 0.4 | 0.3 | 0.09 | 0.15 |
| IQR | 92.65 - 96 | 48.65 - 95.20 | 5.48-25.93 | 10.86-41.55 | 1.58-21.55 | 1.98-25.25 | 3.81 - 4.77 | 4.77 - 6.34 | 1.95-  6.56 | 0.91-5.15 | 0.22-  0.83 | 0.21-0.42 | 0.05-  0.23 | 0.07-0.50 |
| **P value** | *0.1838* | | 0.1419 | | *0.8392* | | *0.1059* | | *0.3736* | | *0.4755* | | *0.5395* | |
| **Innate Immunity PWLH** | | | | | | | | | | | | | | |
|  | NK | | NCM | | CM | | IM | | mDC | | pDC | |  | |
|  | Adenoids | Tonsils | Adenoids | Tonsils | Adenoids | Tonsils | Adenoids | Tonsils | Adenoids | Tonsils | Adenoids | Tonsils |  |  |
| Median | 2.83 | 3.22 | 0.74 | 1.34 | 0.18 | 0.3 | 0.03 | 0.06 | 0.034 | 0.039 | 0.24 | 0.35 |  |  |
| IQR | 1.74-  5.57 | 1.23-7.93 | 0.25-  2.21 | 0.83-2.22 | 0.12-  0.28 | 0.17-0.41 | 0.01-  0.17 | 0.03-  0.11 | 0.02-  0.05 | 0.01-0.08 | 0.16-  0.87 | 0.18-0.48 |  |  |
| **P value** | *0.9654* | | *0.4082* | | *0.1275* | | *0.5553* | | *0.6805* | | *0.984* | |  | |
| **Innate Immunity SN** | | | | | | | | | | | | | | |
|  | NK | | NCM | | CM | | IM | | mDC | | pDC | |  |  |
|  | Adenoids | Tonsils | Adenoids | Tonsils | Adenoids | Tonsils | Adenoids | Tonsils | Adenoids | Tonsils | Adenoids | Tonsils |  |  |
| Median | 6.27 | 8.19 | 0.4 | 2.54 | 0.18 | 0.23 | 0.02 | 0.07 | 0.013 | 0.039 | 0.57 | 0.32 |  |  |
| IQR | 1.60-10.53 | 1.62-17.20 | 0.25-  0.96 | 0.55-5.06 | 0.08-  0.41 | 0.10-0.32 | 0.008-0.04 | 0.01-  0.31 | 0.01-  0.07 | 0.01-0.06 | 0.41-  0.98 | 0.22-0.33 |  |  |
| **P value** | *0.6354* | | *0.0539* | | *0.9999* | | *0.2268* | | *0.7103* | | *0.0539* | |  |  |

Data is shown as median and IQR. Data was compared using the Wilcoxon Rank Sum Test.

Abbreviations: CM: classical macrophages, HIV: human immunodeficiency virus, IQR: interquartile range, IM: inflammatory macrophages, mDC: myeloid dendritic cells, pDC: plasmacytoid dendritic cells, PLWH: people living with HIV, NCM: non-classical macrophages, NK: natural killer, Tfhs: T follicular helper cells, Th: T helper, Treg: T regulatory, SN: seronegative

**Supplementary Figure 3: The proportion of CD4+ and CD8+ T cells, as well as their levels of activation, is similar in adenoids and tonsils**


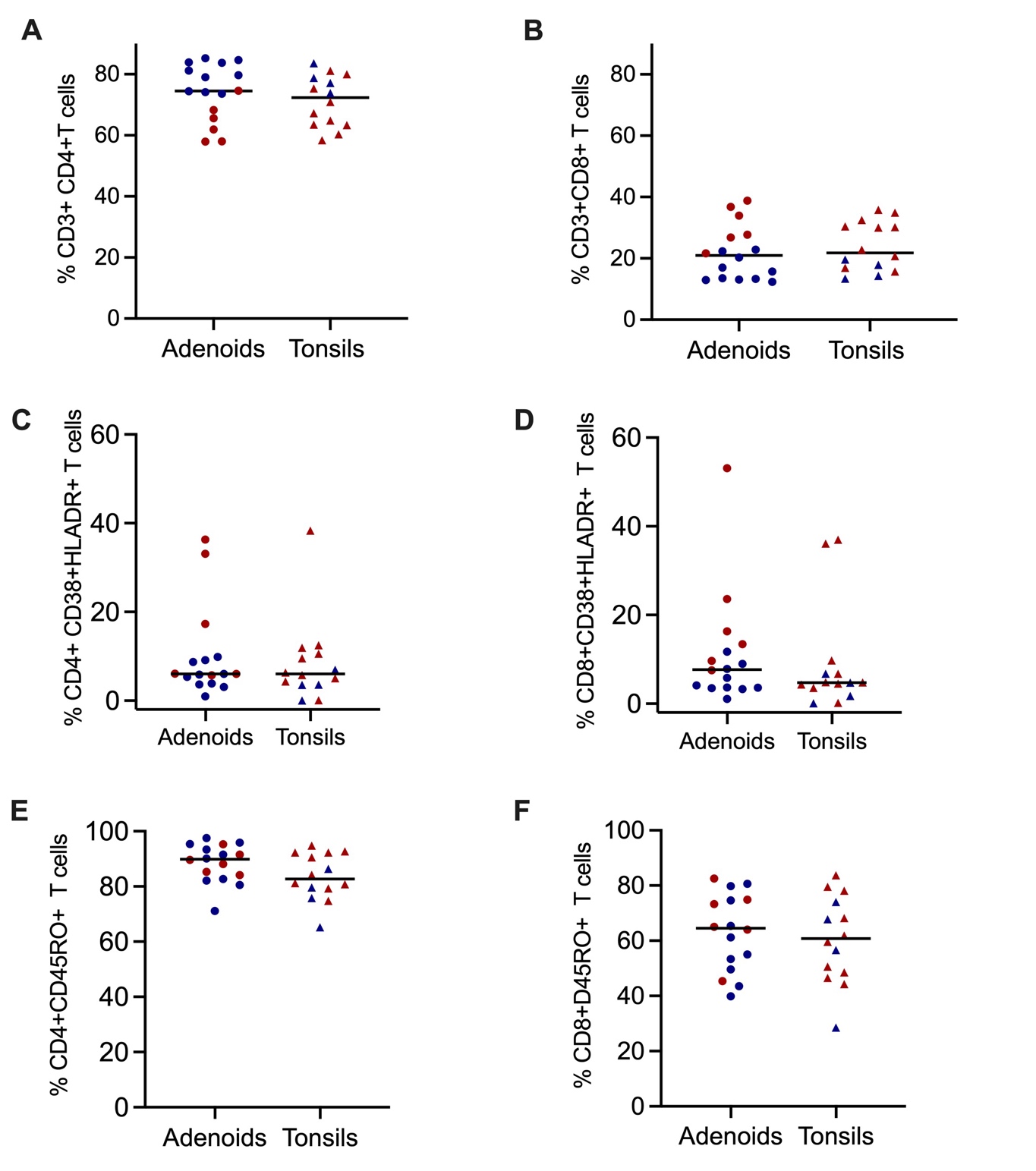


**Legend:** Adenoids (red circle: PLWH, blue circle: SN) and tonsils (red triangle: PLWH, blue triangle: SN) were analyzed for the frequency (%) of CD4+ (A) and CD8+ T cells (B) by flow cytometry. The levels of immune activation were assessed by the co-expression of CD38 and HLADR on CD4+ (C) and CD8+ (D) T cells. The frequency of memory CD4+ (E) and CD8+ T cells (F) defined as CD45RO+ was also analyzed. Scatter plots were used to present the data. The median is shown by a horizontal line. Each symbol represents one individual (red triangle: PLWH tonsils, red circle: PLWH adenoids, blue triangle: SN tonsils, blue circle: SN adenoids). Data was compared using the Wilcoxon Rank Sum Test. Only p values < 0.05 are shown in graphs. *p<0.01; **p<0.001; ***p<0.0001; ****p<0.00001.

Abbreviations: %: frequency, CD: cluster of differentiation, HIV: human immunodeficiency virus, PLWH: people living with HIV, SN: seronegative

**Supplementary Figure 4: PLWH have decreased frequency of CD4+ T cells, increased frequency of CD8+ T cells and increased levels of CD4+ and CD8+ T cell immune activation**


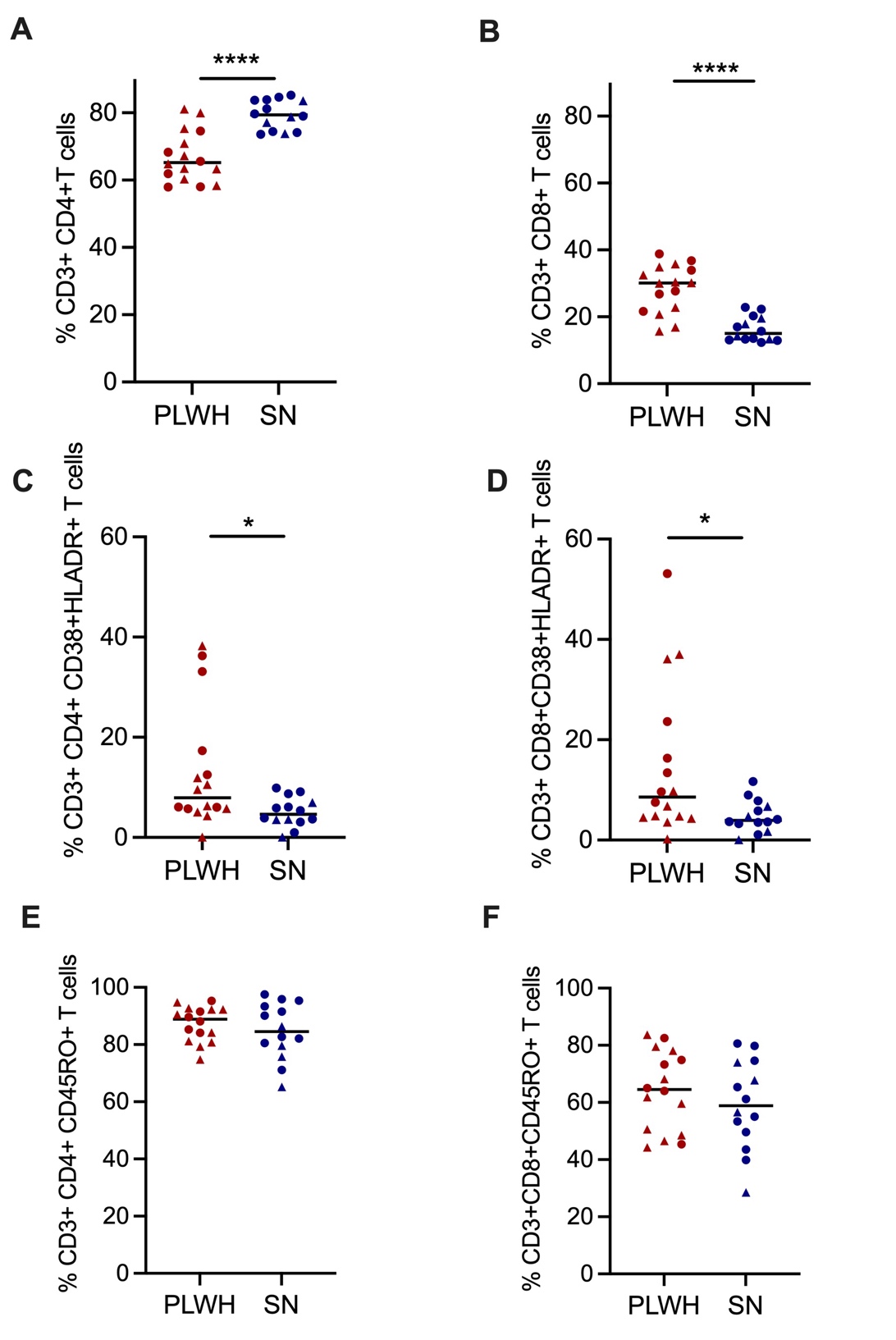


**Legend:** The frequency of CD4+ (A) and CD8+ T cells (B) in the NALT (adenoids and tonsils were pooled together) were analyzed in PLWH (red triangle for tonsils and red circle for adenoids) and SN (blue triangle for tonsils and blue circle for adenoids) individuals. The levels of immune activation were measured by the co-expression of CD38 and HLADR on CD4+ (C) and CD8+ (D) T cells. The frequency of memory CD4+ (E) and CD8+ T cells (F) defined as CD45RO+ were also analyzed. Scatter plots were used to present the data. The median is shown by a horizontal line. Each symbol represents one individual (red triangle: PLWH tonsils, red circle: PLWH adenoids, blue triangle: SN tonsils, blue circle: SN adenoids). PLWH versus SN were compared using the Wilcoxon Rank Sum Test. Only p values < 0.05 are shown in graphs. *p<0.01; **p<0.001; ***p<0.0001; ****p<0.00001.

Abbreviations: %: frequency, CD: cluster of differentiation, HIV: human immunodeficiency virus, NALT: nasopharyngeal associated lymphoid tissue, PLWH: people living with HIV, SN: seronegative

**Supplementary Figure 5: PLWH have similar frequency of B cells and innate immune populations compared to SN**

**A.**

**B**.

**Legend:** Innate immune populations and B cells of the NALT (adenoids and tonsils were pooled together) were analyzed in PLWH (red triangle for tonsils and red circle for adenoids) and SN (blue triangle for tonsils and blue circle for adenoids). A) The frequencies of NK cells (CD56+), macrophages (CD14- CD16+ HLADR-, CD14+ CD16- and CD14+CD16+) and dendritic cells (myeloid dendritic cells CD123-CD11c+ and plasmacytoid dendritic cells CD123+ CD11c-) are shown. B) The frequency of B cells (CD19+) is shown. Scatter plots were used to present the data. The median is shown by a horizontal line. Each symbol represents one individual. PLWH versus SN were compared using the Wilcoxon Rank Sum Test. Only p values < 0.05 are shown in graphs. *p<0.01; **p<0.001; ***p<0.0001; ****p<0.00001.

Abbreviations: %: frequency, CD: cluster of differentiation, CM: classical macrophages, HIV: human immunodeficiency virus, IM: inflammatory macrophages, mDC: myeloid dendritic cells, NCM: non-classical macrophages, pDC: plasmacytoid dendritic cells, PLWH: people living with HIV, SN: seronegative

**Supplementary Table 4: Demographic and clinical characteristics of individuals with nasopharyngeal 16S data (n=9)**

| Sample | Group | Gender | Age | ART | CD4 | CD4/CD8 | pVL | Abx | Residence | Oral diseases | Flossing | Mouthwash | Smoking |
| --- | --- | --- | --- | --- | --- | --- | --- | --- | --- | --- | --- | --- | --- |
| S062 | SN | M | 20 | NA | 519 | 1.3 | NA | U | CDMX | U | NO | YES | NO |
| S065 | SN | M | 23 | NA | 1298 | 1.58 | NA | NO | CDMX | Caries | NO | Sometimes | NO |
| S052 | PLWH | M | 27 | PI | 322 | 0.52 | <40 | U | OTHERS | NO | NO | YES | NO |
| S054 | PLWH | M | 38 | NNRTI | 947 | 1.02 | <40 | NO | CDMX | NO | YES | YES | NO |
| S055 | PLWH | M | 32 | NNRTI | 1136 | 0.94 | <40 | U | CDMX | Gingivitis/Caries | YES | NO | NO |
| S056 | PLWH | M | 26 | NNRTI | 703 | 0.71 | 47 | NO | EDOMEX | Caries | YES | NO | NO |
| S057 | PLWH | M | 32 | PI | 743 | 1.21 | <40 | U | CDMX | U | U | U | U |
| S058 | PLWH | M | 38 | NNRTI | 1200 | 0.94 | 51 | NO | EDOMEX | Gingivitis/Caries | NO | NO | Yes |
| S060 | PLWH | M | 39 | NNRTI | 1002 | 0.99 | <40 | NO | CDMX | NO | YES | YES | NO |

| Sample | Days since HIV Dx | Days on ART |
| --- | --- | --- |
| S062 | NA | NA |
| S065 | NA | NA |
| S052 | 1052 | 1037 |
| S054 | 4093 | 1457 |
| S055 | 2620 | 2284 |
| S056 | 1128 | 1105 |
| S057 | 621 | 593 |
| S058 | 3629 | 3550 |
| S060 | 4471 | 1835 |

Abbreviations: Abx: antibiotics, ART: antiretroviral, CDMX: Mexico City, CD4: CD4 T cell count (cells/mm^3^), Dx: diagnosis, EDOMEX: State of Mexico, HIV: human immunodeficiency virus, M: man, NA: not applicable, NNRTI: non-nucleoside reverse transcriptase inhibitors, PLWH: people living with HIV, PI: protease inhibitors, SN: seronegative, U: unknown

**Supplementary Table 5: Demographic and clinical characteristics of individuals with tonsillar and oral 16S data (n=16)**

| Individual | Group | ART | Oral wash | Oral  ID | Oropharynx Samples | | | | Gender | Age | CD4 | CD4/CD8 | pVL | Abx | Residence |
| --- | --- | --- | --- | --- | --- | --- | --- | --- | --- | --- | --- | --- | --- | --- | --- |
|  |  |  |  |  | LATP | TF-L | RATP | TF-R |  |  |  |  |  |  |  |
| 1 | PLWH | NNRTI | NO | NA | S001 | S003 | NA | S002 | M | 28 | 412 | 0.35 | 524 | U | CDMX |
| 2 | PLWH | NNRTI | NO | NA | S005 | S007 | S004 | S006 | M | 33 | 346 | 0.24 | <40 | NO | CDMX |
| 3 | PLWH | NNRTI | YES | S066 | S009 | S011 | S008 | S010 | M | 32 | 1136 | 0.94 | <40 | U | CDMX |
| 4 | PLWH | NNRTI | NO | NA | S013 | S015 | S012 | S014 | M | 32 | 538 | 0.88 | <40 | NO | CDMX |
| 5 | PLWH | NNRTI | YES | S067 | S017 | S019 | S016 | S018 | M | 32 | 497 | 0.65 | <40 | NO | CDMX |
| 6 | PLWH | NNRTI | YES | S068 | NA | S026 | S023 | S025 | M | 51 | 211 | 0.74 | <40 | NO | CDMX |
| 7 | PLWH | INSTI | YES | S070 | S032 | S034 | S031 | S033 | M | 25 | 601 | 0.68 | 427541 | NO | CDMX |
| 8 | PLWH | ART | YES | S071 | S036 | NA | S035 | NA | M | 24 | 584 | 0.71 | <40 | U | U |
| 9 | PLWH | ART | NO | NA | NA | NA | S042 | NA | M | 34 | 939 | 1.05 | <40 | YES | EDOMEX |
| 10 | PLWH | ART | YES | S074 | NA | NA | NA | NA | M | 19 | 1522 | 0.89 | <40 | NO | CDMX |
| 11 | PLWH | ART | YES | S076 | NA | NA | NA | NA | M | 29 | 849 | 0.9 | <40 | YES | CDMX |
| 12 | SN | NA | NO | NA | NA | NA | S051 | NA | F | 32 | 917 | 1.07 | NA | NO | CDMX |
| 13 | SN | NA | YES | S069 | S028 | S030 | S027 | S029 | M | 32 | 1240 | 1.67 | NA | NO | CDMX |
| 14 | SN | NA | YES | S072 | S043 | NA | NA | NA | F | 27 | 1216 | 2.31 | NA | NO | EDOMEX |
| 15 | SN | NA | NO | NA | S045 | NA | NA | NA | M | 35 | 1461 | 1.6 | NA | YES | CDMX |
| 16 | SN | NA | YES | S075 | S049 | S050 | NA | NA | F | 34 | 1213 | 1.81 | NA | NO | EDOMEX |

| Individual | Days HIV Dx | Days on ART | Oral diseases | Flossing | Mouthwash | Smoking |
| --- | --- | --- | --- | --- | --- | --- |
| 1 | 88 | 35 | U | NO | Sometimes | U |
| 2 | 1219 | 1176 | Gingivitis | NO | YES | NO |
| 3 | 2620 | 2284 | Caries/Gingivitis | YES | NO | NO |
| 4 | 1171 | 1098 | Caries | YES | YES | YES |
| 5 | 3143 | 2833 | Caries/Gingivitis/Stomatitis | NO | YES | NO |
| 6 | 3124 | U | Caries/Gingivitis | YES | YES | NO (Former smoker) |
| 7 | 28 | 2 | Caries/Gingivitis | NO | NO | NO |
| 8 | U | U | U | U | U | U |
| 9 | 3185 | U | Caries | YES | YES | YES |
| 10 | U | U | Caries | NO | NO | NO (Former smoker) |
| 11 | U | U | NO | YES | Sometimes | YES |
| 12 | NA | NA | Caries | NO | Sometimes | NO |
| 13 | NA | NA | Caries | YES | NO | YES |
| 14 | NA | NA | Caries/Gingivitis | NO | Sometimes | NO |
| 15 | NA | NA | NO | Sometimes | Sometimes | NO |
| 16 | NA | NA | NO | NO | Sometimes | YES |

Abbreviations: Abx: antibiotics, ART: antiretroviral, CDMX: Mexico City, CD4: CD4 T cell count (cells/mm^3^), Dx: diagnosis, EDOMEX: State of Mexico, HIV: human immunodeficiency virus, ID: identifier, LATP: left anterior tonsillar pillar, M: man, NA: not applicable, NNRTI: non-nucleoside reverse transcriptase inhibitors, PLWH: people living with HIV, OTHERS: neither CDMX or EDOMEX, PI: protease inhibitors, RATP: right anterior tonsillar pillar, S: Samples, SN: seronegative, TF-L: tonsillar fossa-left, TF-R: tonsillar fossa-right, U: unknown

**Supplementary Table 6: Mean Relative Abundance (%) of the top 5 phyla and 10 genera in the nasopharynx of PLWH and SN**

1. **Mean Relative Abundance (%) of the top 5 phyla**

| Phylum | Overall | PLWH | SN | P |
| --- | --- | --- | --- | --- |
| Firmicutes | 79.07 | 86.12 | 54.38 | 0.11 |
| Actinobacteria | 17.94 | 12.78 | 36.01 | 0.33 |
| Proteobacteria | 2.77 | 0.81 | 9.61 | 1 |
| Bacteroidetes | 0.21 | 0.28 | 0.0 | 0.26 |
| Fusobacteria | 0.01 | 0.01 | 0.0 | 0.79 |
| Others | 0.0 | 0.0 | 0.7 | NA |

1. **Mean Relative Abundance (%) of the top 10 genera**

| Phylum | Genus | Overall | PLWH | SN | P |
| --- | --- | --- | --- | --- | --- |
| Firmicutes | *Staphylococcus* | 72.8 | 80.6 | 45.6 | 0.11 |
| Actinobacteria | *Lawsonella* | 8.2 | 6.8 | 13.0 | 1 |
| Actinobacteria | *Corynebacterium* | 7.4 | 3.6 | 21.0 | 0.11 |
| Firmicutes | *Streptococcus* | 3.3 | 3.0 | 4.6 | 1 |
| Firmicutes | *Finegoldia* | 2.9 | 2.5 | 4.2 | 0.46 |
| Actinobacteria | *Cutibacterium* | 2.3 | 2.3 | 2.0 | 0.89 |
| Proteobacteria | *Haemophilus* | 1.0 | 0.2 | 3.7 | 0.63 |
| Proteobacteria | *Neisseria* | 0.9 | 0.0 | 3.9 | 0.32 |
| Proteobacteria | *Kluyvera* | 0.5 | 0.1 | 2.0 | 0.32 |
| Proteobacteria | *Ochrobactrum* | 0.2 | 0.3 | 0.0 | 0.1 |
|  | Others | 0.5 | 0.6 | 0.0 | NA |

Mean relative abundances were compared between PLWH and SN using the Wilcoxon Rank Sum test.

Abbreviations: %: percentage, HIV: human immunodeficiency virus, PLWH: people living with HIV, NA: not applicable, P: p value, SN: seronegative

**Supplementary Figure 6: Taxa barplots at phylum level, and boxplots comparing the nasopharyngeal microbiota of PLWH with SN**


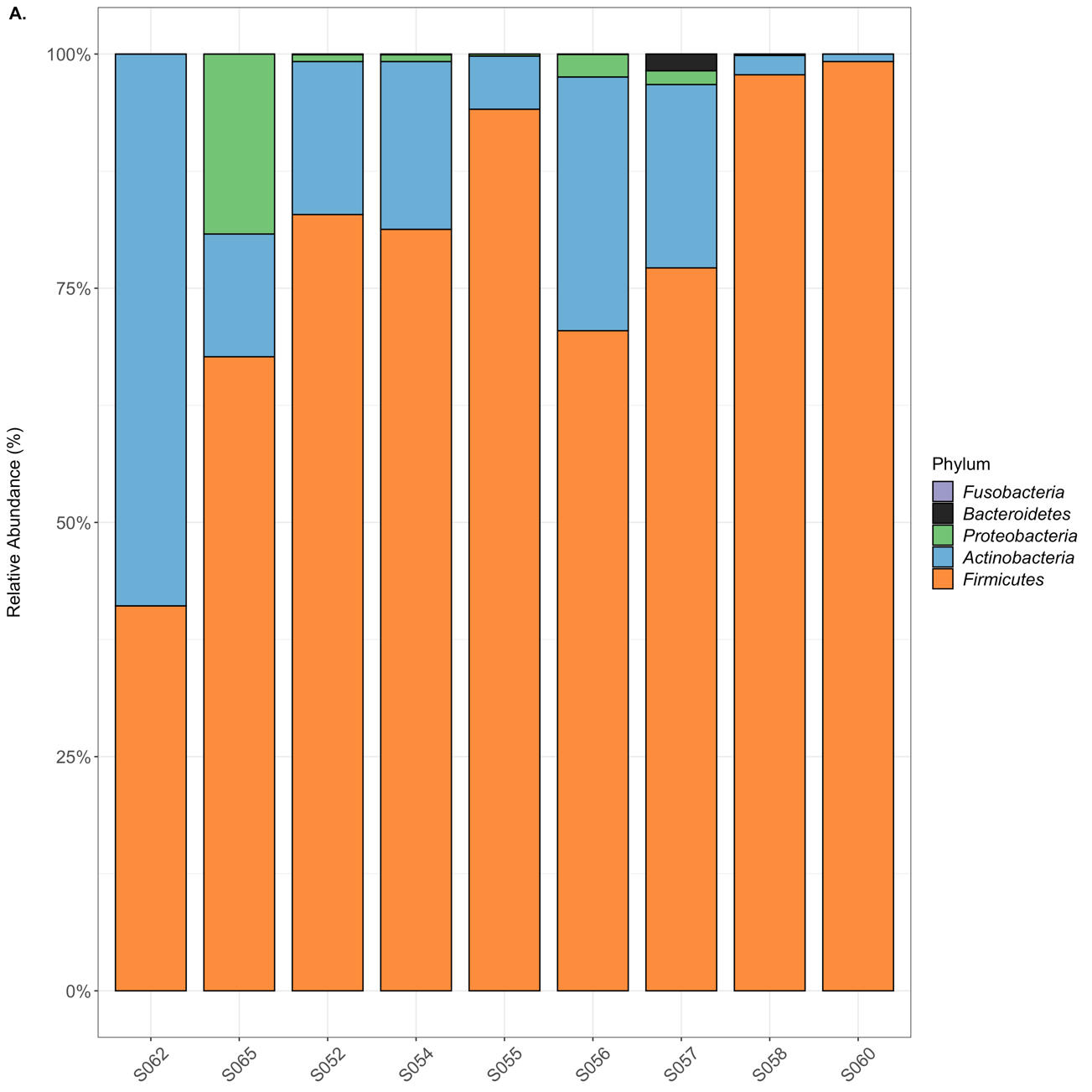


**
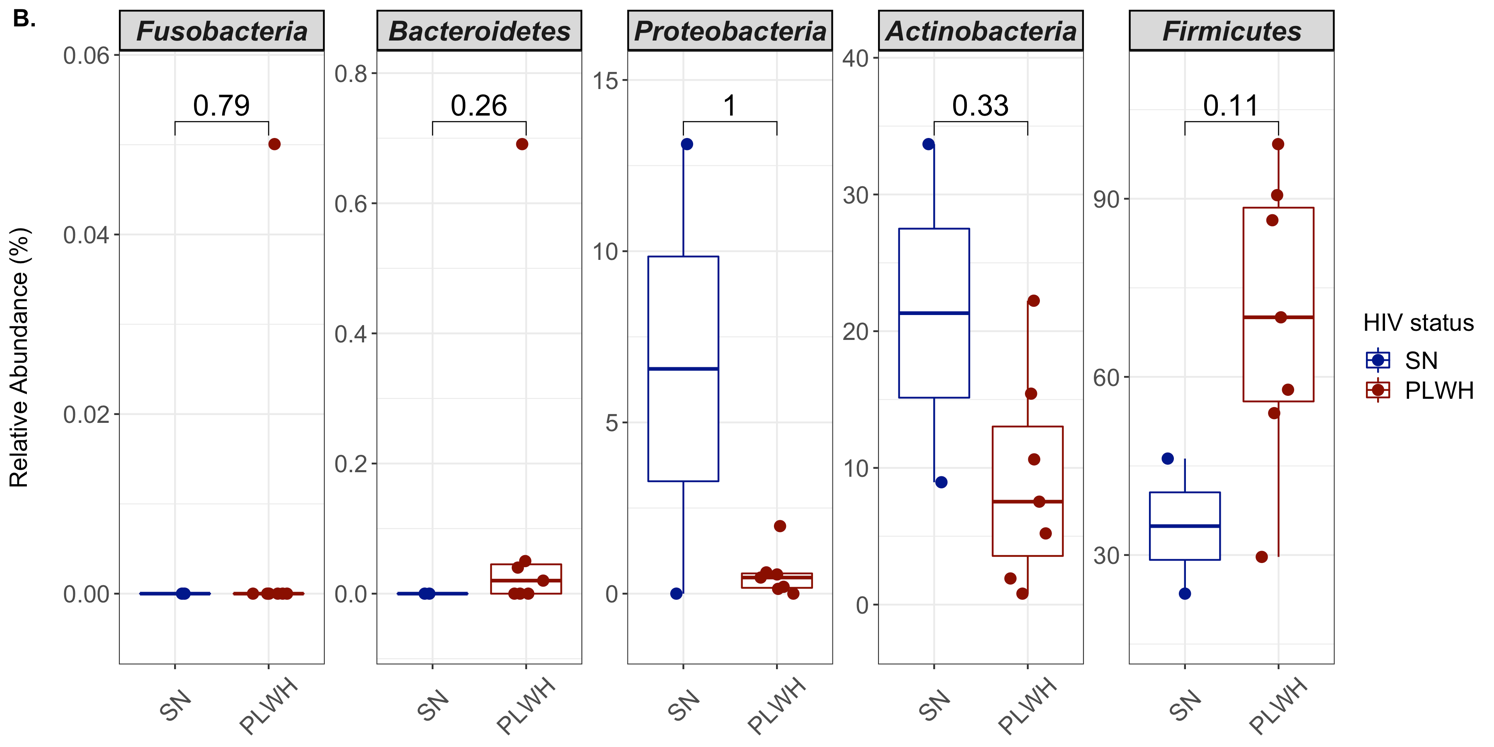
**

**Legend:**

A. Taxa barplots showing the bacterial composition at phylum level. Phyla are listed in ascending relative abundance (*Fusobacteria*, *Bacteroidetes*, *Proteobacteria*, *Actinobacteria*, *Firmicutes*). Each barplot corresponds to one individual: SN: S062, S065; PLWH: S052, S054, S055, S056, S057, S058 and S060

B. Boxplots depicting the median and interquartile range. The relative abundance of each phylum was compared between PLWH and SN using the Wilcoxon Rank Sum test. There were no differences between these two groups.

Abbreviations: %: percentage, HIV: human immunodeficiency virus, PLWH: people living with HIV, SN: seronegative

**Supplementary Figure 7: Comparison of the top 10 genera of the nasopharyngeal microbiota between PLWH and SN**


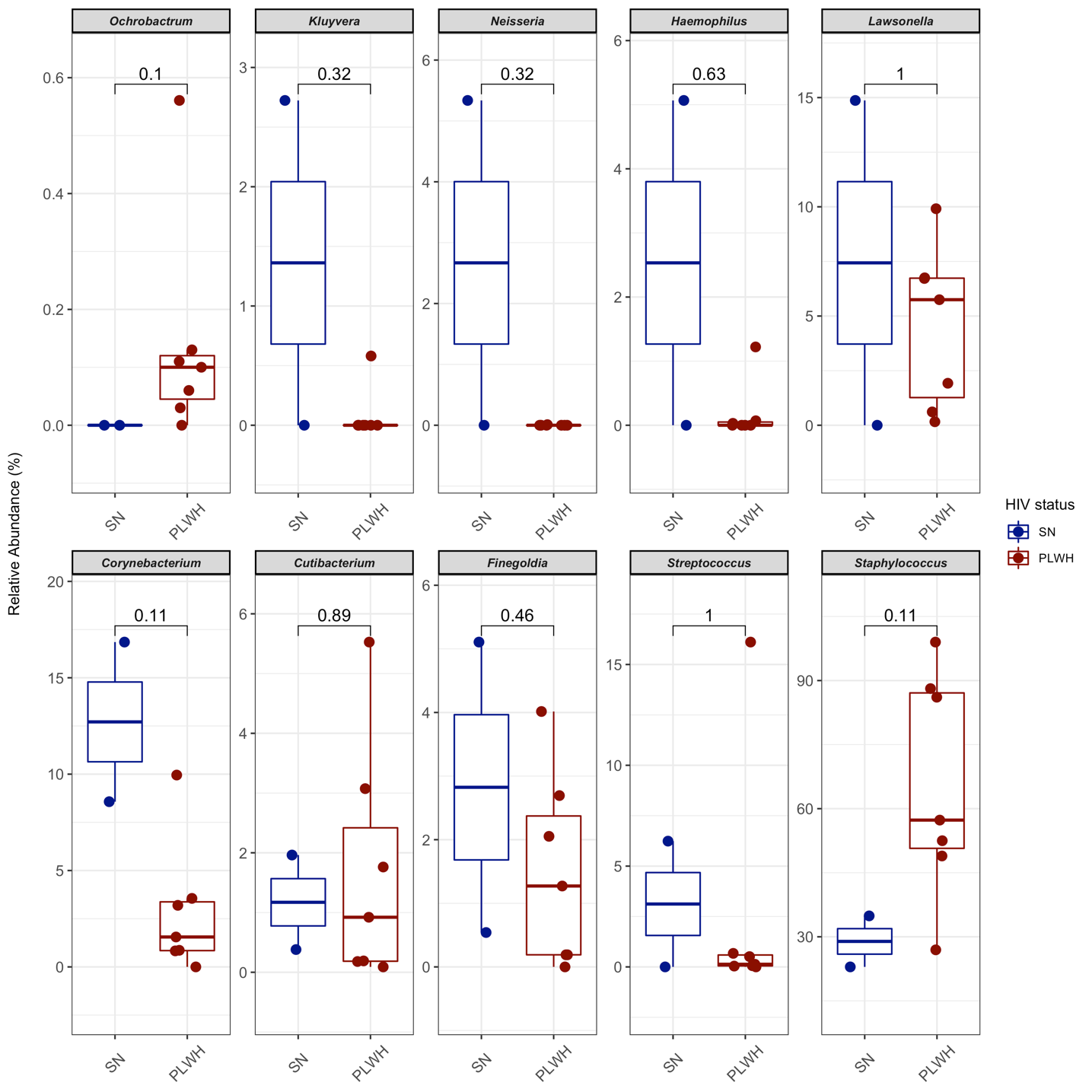


**Legend:**

Boxplots showing the median and interquartile range. The relative abundance of the top 10 genera was compared between PLWH and SN using the Wilcoxon Rank Sum test. There were no differences between these two groups. The top 10 genera belong to 3 phyla: Proteobacteria (*Ochrobactrum*, *Kluyvera*, *Neisseria*, *Haemophilus*), Actinobacteria (*Lawsonella*, *Corynebacterium*, *Cutibacterium*) and Firmicutes (*Finegoldia*, *Streptococcus*, *Staphylococcus*).

Abbreviations: %: percentage, HIV: human immunodeficiency virus, PLWH: people living with HIV, SN: seronegative

**Supplementary Table 7: Alpha diversity at each anatomical site of the oropharynx stratified by HIV status**

|  |  | Richness | | Shannon | |
| --- | --- | --- | --- | --- | --- |
|  | n | Median [IQR] | p | Median [IQR] | p |
| Oral Rinse | 7 PLWH  3 SN | 202 [132-280]  277 [203-291] | 0.26 | 3.561 [3.476-3.757]  3.720 [3.579-4.063] | 0.26 |
| Oropharynx | 11 PLWH  5 SN | 102 [34-155]  105 [42-196.5] | 0.704 | 3.076 [2.401-3.623]  3.172 [2.651-4.128] | 0.22 |
| LATP | 7 PLWH  4 SN | 86 [28-161]  127.5 [71.25-223.5] | 0.41 | 2.697 [2.482-4.033]  3.483 [2.807-4.194] | 0.41 |
| RATP | 8 PLWH  2 SN | 110 [40.25-150]  88.50 [46-131] | 0.71 | 3.072 [2.213-4.013]  3.273 [2.617-3.929] | 0.88 |
| TF-L | 7 PLWH  2 SN | 158 [30-174]  133.5 [24-243] | 0.88 | 3.124 [2.132-3.620]  3.536 [2.744-4.327] | 0.50 |
| TF_R | 7 PLWH  1 SN | 102 [37-148]  38 | NA | 3.076 [2.270-3.434]  2.289 | NA |

Data is shown as median and interquartile range [IQR]. The number of PWLH and SN with 16S data at each anatomical site is shown. Alpha diversity metrics were compared between PLWH and SN using the Wilcoxon Rank Sum test

Abbreviations:

HIV: human immunodeficiency virus, LATP: left anterior tonsillar pillar, n: number, NA: not applicable, PLWH: people living with HIV, RATP: right anterior tonsillar pillar, SN: seronegative, TF-L: tonsillar fossa-left, TF-R: tonsillar fossa-right

**Supplementary Figure 8: Alpha diversity (A. Richness, B. Shannon) is similar in the oropharynx region and the oral cavity between PLWH and SN**


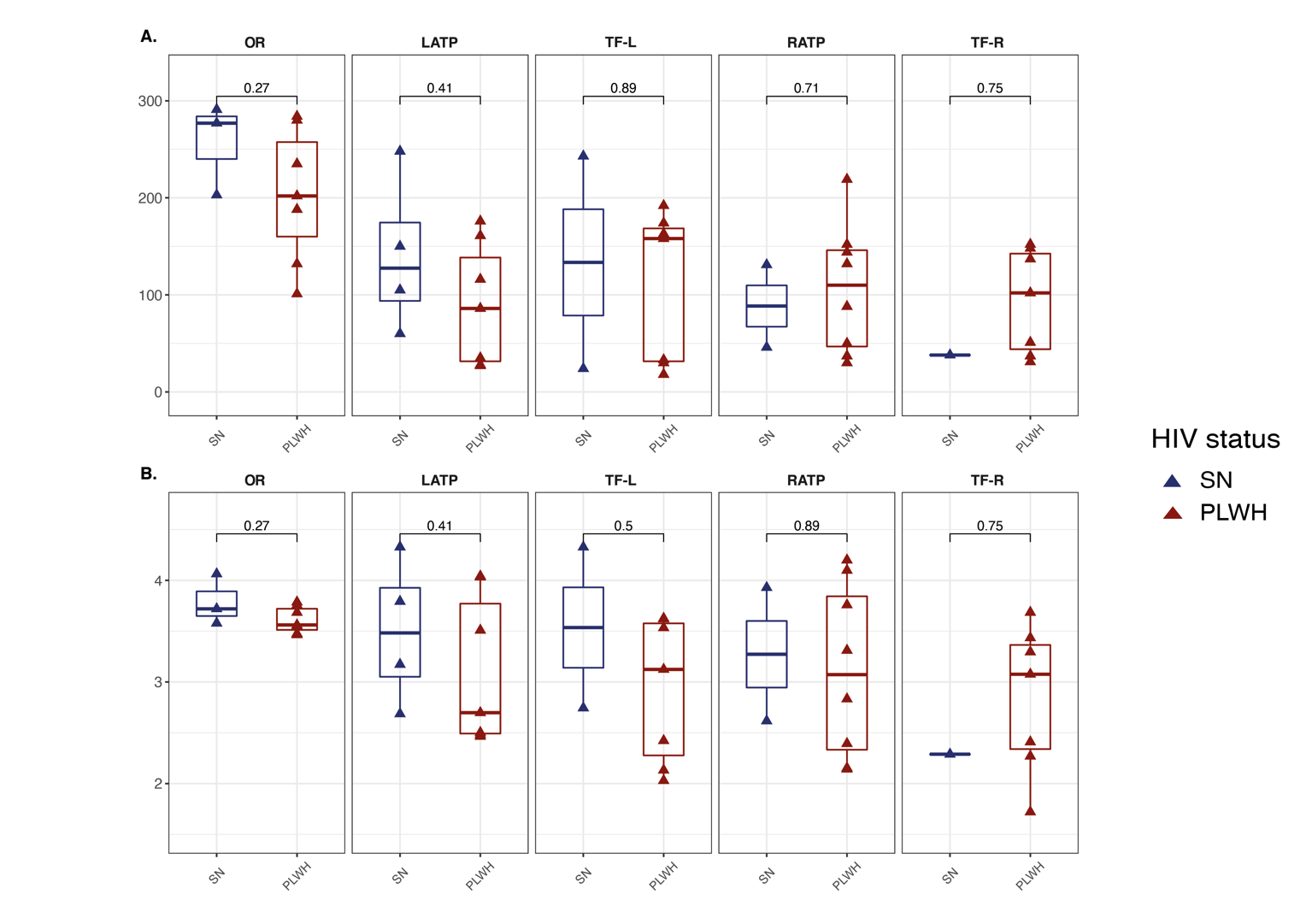


**Legend:**

Boxplots showing the median and interquartile range. Two alpha metrics were calculated: A. Richness (observed species) and B. shannon, and compared between PLWH and SN at the 4 tonsillar sites: left and right anterior tonsillar pillar (LATP and RATP, respectively) and left and right tonsillar fossa (TF-L and TF-R, respectively), and oral rinse (OR) using the Wilcoxon Rank Sum test. There were no differences between these two groups.

Abbreviations: HIV: human immunodeficiency virus, LATP: left anterior tonsillar pillar, PLWH: people living with HIV, SN: seronegative, RATP: right anterior tonsillar pillar, TF-L: tonsillar fossa-left, TF-R: tonsillar fossa-right

**Supplementary Table 8: R squared and PERMANOVA p for each anatomical site of the oropharynx region and the oral cavity**

|  |  |  | Bray-Curtis | |
| --- | --- | --- | --- | --- |
|  | n | Df | R2 | PERMANOVA P |
| Oral Rinse | 7 PLWH  3 SN | 1 | 0.089 | 0.762 |
| Oropharynx | 11 PLWH  5 SN | 1 | 0.034 | 0.154 |
| LATP | 7 PLWH  4 SN | 1 | 0.081 | 0.842 |
| RATP | 8 PLWH  2 SN | 1 | 0.115 | 0.297 |
| TF-L | 7 PLWH  2 SN | 1 | 0.113 | 0.539 |
| TF-R | 7 PLWH  1 SN | 1 | 0.120 | 0.598 |

The number of PWLH and SN with 16S data for each anatomical site is shown.

Permutation test for homogeneity of multivariate dispersions was checked prior to running adonis using betadisper function and subjected to ANOVA.

Abbreviations: Df: degrees of freedom, HIV: human immunodeficiency virus, LATP: left anterior tonsillar pillar, n: number, PERMANOVA: Permutational multivariate analysis of variance, PLWH: people living with HIV, RATP: right anterior tonsillar pillar, R2: R squared, SN: seronegative, TF-L: tonsillar fossa-left, TF-R: tonsillar fossa-right

**Supplementary Table 9: Mean Relative Abundance (%) of the top 6 phyla and the top 20 genera in the oropharynx and oral cavity of PLWH and SN**

1. **Mean Relative Abundance (%) of the top 6 phyla**

| Phylum | Overall | PLWH | SN | OR | Oropharynx^¥^ |
| --- | --- | --- | --- | --- | --- |
| Firmicutes | 45.5 | 45.7 | 44.7 | 45.7 | 45.7 |
| Bacteroidetes | 19.0 | 18.7 | 19.9 | 18.7 | 18.7 |
| Proteobacteria | 15.5 | 15.4 | 17.8 | 15.4 | 15.4 |
| Fusobacteria | 14.3 | 14.7 | 10.9 | 14.7 | 14.7 |
| Actinobacteria | 4.1 | 4.1 | 4.1 | 4.1 | 4.1 |
| Spirochetes | 1.3 | 1.0 | 1.9 | 1.0 | 1.0 |
| Others | 0.3 | 0.4 | 0.7 | 0.4 | 0.4 |

1. **Mean Relative Abundance (%) of the top 20 genera**

| Phylum | Genus | Overall | PLWH | SN | OR | Oropharynx^¥^ |
| --- | --- | --- | --- | --- | --- | --- |
| Firmicutes | *Streptococcus* | 26.21 | 25.76 | 27.59 | 26.21 | 26.21 |
| Fusobacterium | *Fusobacterium* | 11.48 | 11.91 | 10.19 | 11.48 | 11.48 |
| Bacteroidetes | *Prevotella* | 10.16 | 10.96 | 8.46 | 10.16 | 10.16 |
| Proteobacteria | *Haemophilus* | 8.79 | 8.92 | 8.39 | 8.79 | 8.79 |
| Firmicutes | *Veillonella* | 6.58 | 7.39 | 7.74 | 6.58 | 6.58 |
| Firmicutes | *Porphyromonas* | 5.96 | 6.08 | 5.64 | 5.96 | 5.96 |
| Proteobacteria | *Neisseria* | 4.28 | 2.89 | 5.61 | 4.28 | 4.28 |
| Firmicutes | *Parvimonas* | 2.19 | 2.20 | 4.16 | 2.19 | 2.19 |
| Actinobacteria | *Rothia* | 1.93 | 2.10 | 2.14 | 1.93 | 1.93 |
| Bacteroidetes | *Alloprevotella* | 1.87 | 1.93 | 1.95 | 1.87 | 1.87 |
| Fusobacteria | *Sneathia* | 1.61 | 1.59 | 1.93 | 1.61 | 1.61 |
| Firmicutes | *Peptostreptococcus* | 1.48 | 1.54 | 1.43 | 1.48 | 1.48 |
| Actinobacteria | *Actinomyces* | 1.28 | 1.41 | 1.31 | 1.28 | 1.28 |
| Spirochaetes | *Treponema* | 1.25 | 1.32 | 1.22 | 1.25 | 1.25 |
| Fusobacteria | *Leptotrichia* | 1.21 | 1.08 | 1.17 | 1.21 | 1.21 |
| Proteobacteria | *Aggregatibacter* | 1.19 | 1.05 | 1.16 | 1.19 | 1.19 |
| Firmicutes | *Granulicatella* | 1.17 | 1.03 | 0.87 | 1.17 | 1.17 |
| Firmicutes | *Gemella* | 1.12 | 0.97 | 0.79 | 1.12 | 1.12 |
| Firmicutes | *Filifactor* | 0.87 | 0.75 | 0.73 | 0.87 | 0.87 |
| Proteobacteria | *Campylobacter* | 0.86 | 0.63 | 0.72 | 0.86 | 0.86 |
| Others | Others | 8.51 | 8.49 | 6.8 | 8.51 | 8.51 |

^¥^The oropharynx is comprised of the left anterior tonsillar pillar, the right anterior tonsillar pillar, the left tonsillar fossa and the right tonsillar fossa.

Abbreviations: %: percentage, HIV: human immunodeficiency virus, PLWH: people living with HIV, SN: seronegative, OR: oral rinse

**Supplementary Figure 9: Comparison of the mean relative abundance of the top 6 phyla in the oral cavity and oropharynx between PLWH and SN**


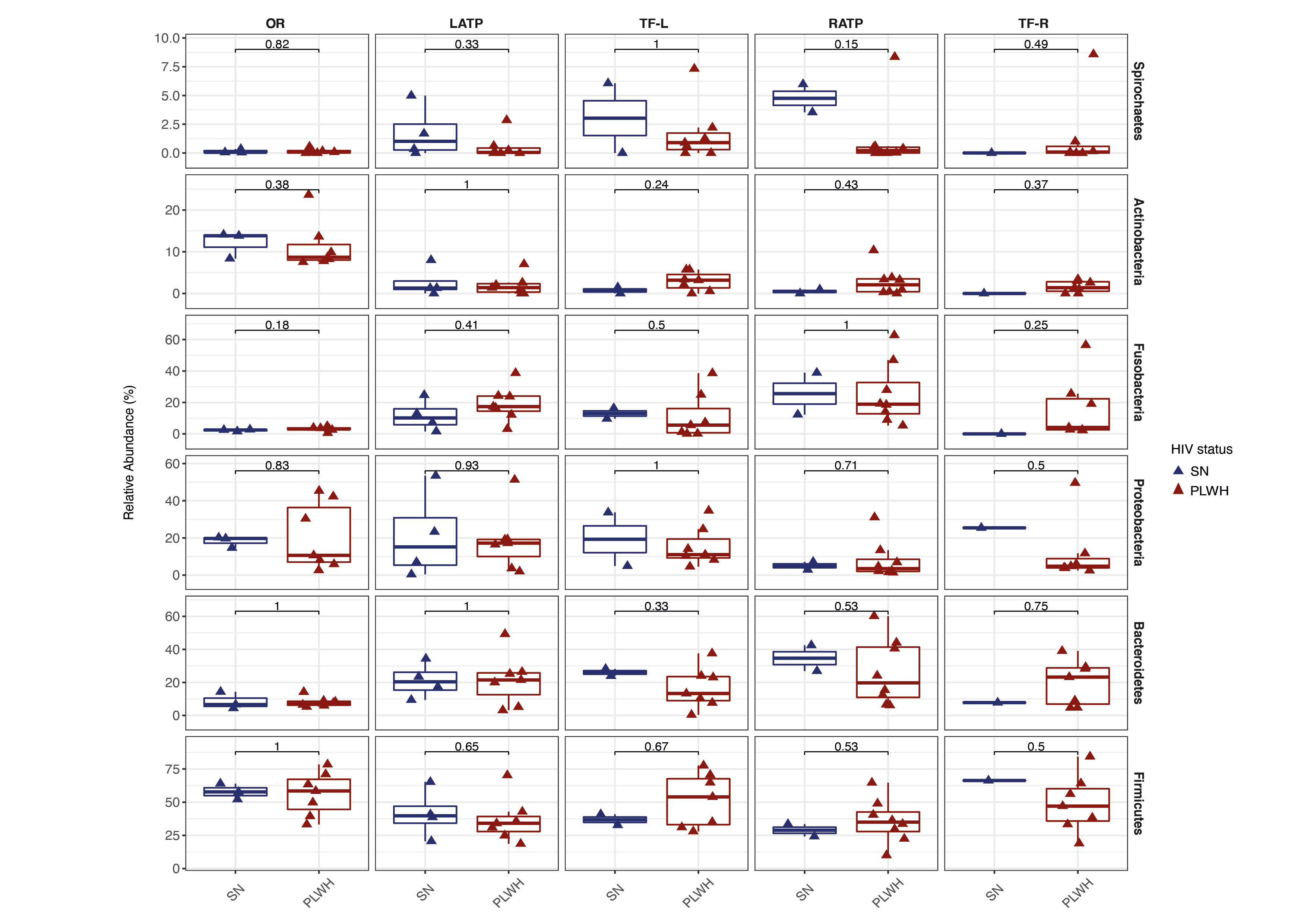


**Legend:** Boxplots showing the median and interquartile range. The relative abundance of the top 6 phyla were compared between PLWH and SN at the 4 tonsillar sites: left and right anterior tonsillar pillar (LATP and RATP, respectively) and left and right tonsillar fossa (TF-L and TF-R, respectively), and oral rinse (OR) using the Wilcoxon Rank Sum test. There were no differences between PLWH and SN.

Abbreviations: %: percentage, HIV: human immunodeficiency virus, LATP: left anterior tonsillar pillar, OR: oral rinse, PLWH: people living with HIV, RATP: right anterior tonsillar pillar, SN: seronegative, TF-L: tonsillar fossa-left, TF-R: tonsillar fossa-right

**Supplementary Table 10: Samples with 16S rRNA sequences at baseline and at follow-up for the oropharyngeal region**

|  | **PLWH1** | | **PLWH2** | | **SN1** | | **SN2** | |
| --- | --- | --- | --- | --- | --- | --- | --- | --- |
|  | **Baseline** | **Follow-up** | **Baseline** | **Follow-up** | **Baseline** | **Follow-up** | **Baseline** | **Follow-up** |
| **LATP** | S017 | S021 | S036 | S038 | S043 | NA | S045 | NA |
| **TF-L** | S019 | NA | NA | S039 | NA | NA | NA | S047 |
| **RATP** | S016 | S020 | S035 | S037 | NA | NA | NA | S046 |
| **TF-R** | S018 | S022 | NA | NA | NA | S044 | NA | NA |

Swabs were obtained from all 4 sites at both baseline and follow-up (6 months after tonsillectomy), NA signifies that swabs did not amplify or/and too little 16S rDNA sequences were obtained after quality-control and dada2.

Abbreviations: HIV: human immunodeficiency virus, LATP: left anterior tonsillar pillar, PLWH: people living with HIV, RATP: right anterior tonsillar pillar, SN: seronegative, TF-L: tonsillar fossa-left, TF-R: tonsillar fossa-right¡

**Supplementary Figure 10:** **Taxa barplots at phylum level at baseline and at follow-up (after tonsillectomy) faceted by each subject**


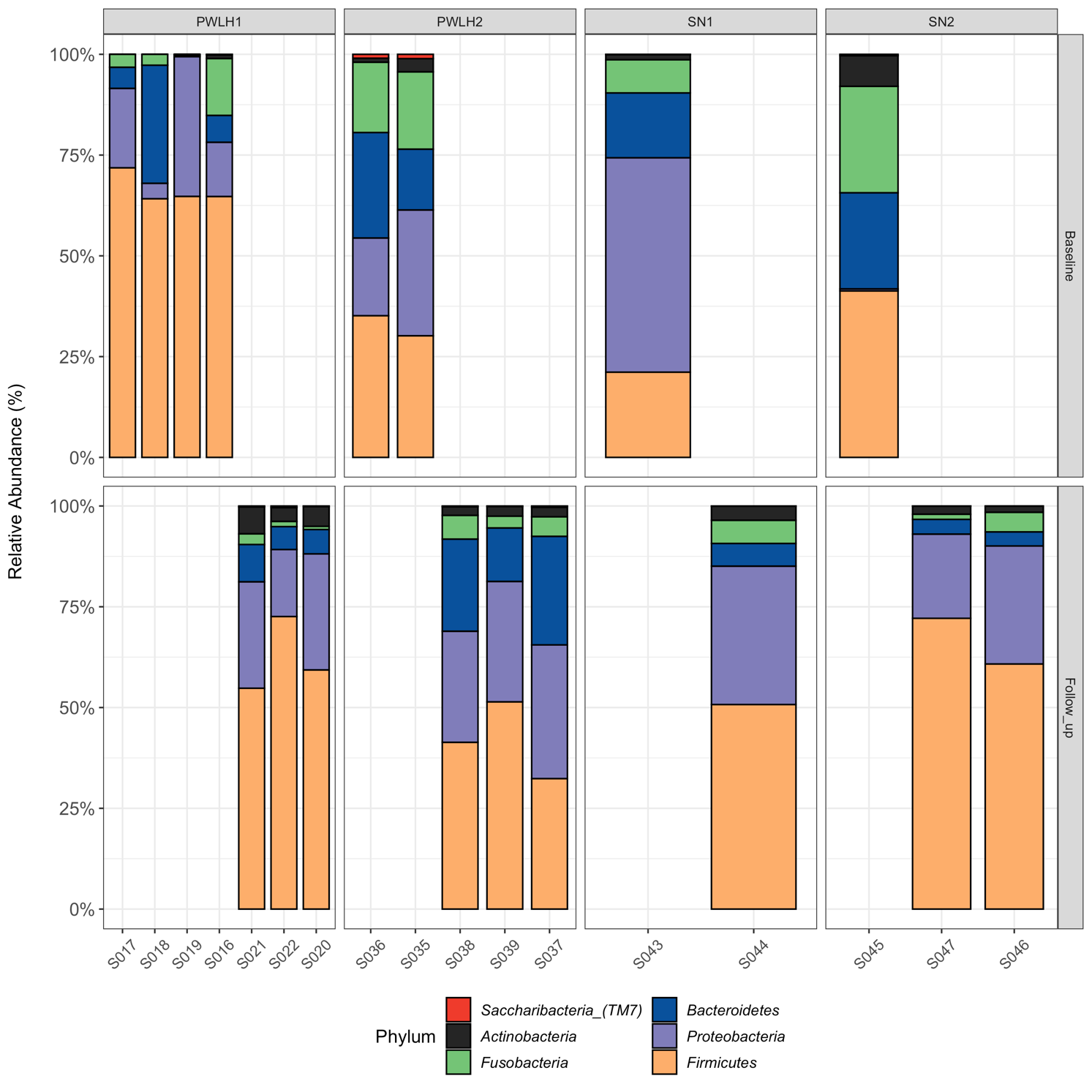


**Legend:** Taxa barplots showing the bacterial composition at phylum level faceted by HIV status and pre- and post-tonsillectomy. Phyla are listed in ascending relative abundance. Each barplot corresponds to one individual and one anatomical site.

PLWH1: At baseline: S017: LATP, S018: TF-R, S019: TF-L, S016: RATP; at follow-up: S021: LATP, S022: TF-R, and S020: RATP

PLWH2: At baseline: S036: LATP, S035: RATP; at follow-up: S038: LATP, S039: TF-L, S037: RATP

SN1: At baseline: S043: LATP; at follow-up: S044: TF-R

SN2: At baseline: S045: LATP; at follow-up: S047: TF-L, S046: RATP

Abbreviations: %: percentage, HIV: human immunodeficiency virus, LATP: left anterior tonsillar pillar, OR: oral rinse, PLWH: people living with HIV, RATP: right anterior tonsillar pillar, SN: seronegative, TF-L: tonsillar fossa-left, TF-R: tonsillar fossa-right

**Supplementary Figure 11:** **Taxa barplots at genus level at baseline and at follow-up (after tonsillectomy) faceted by each subject**


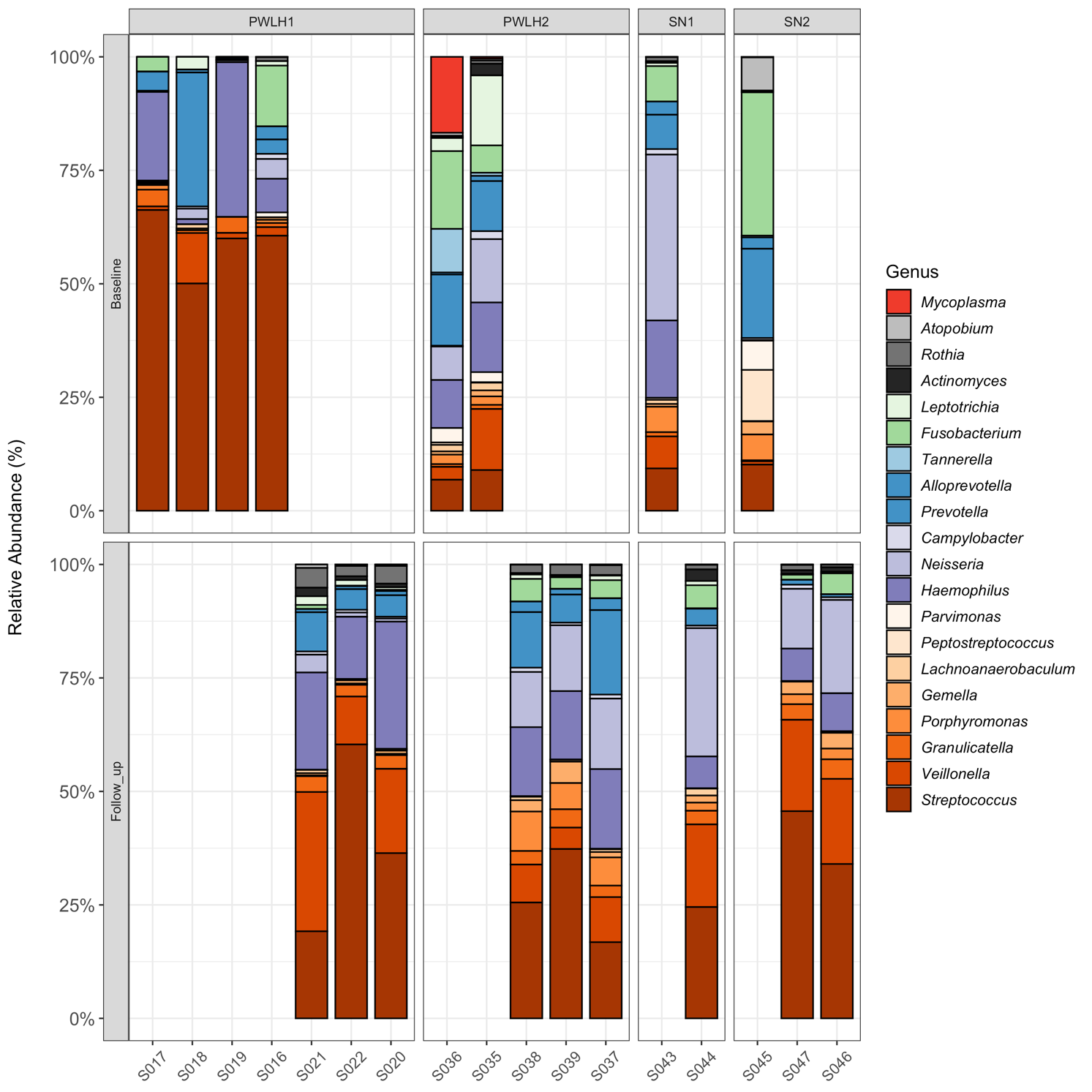


**Legend:** Taxa barplots showing the bacterial composition at genus level faceted by HIV status and pre- and post-tonsillectomy. Each barplot corresponds to one individual and one anatomical site.

PLWH1: At baseline: S017: LATP, S018: TF-R, S019: TF-L, S016: RATP; at follow-up: S021: LATP, S022: TF-R, and S020: RATP

PLWH2: At baseline: S036: LATP, S035: RATP; at follow-up: S038: LATP, S039: TF-L, S037: RATP

SN1: At baseline: S043: LATP; at follow-up: S044: TF-R

SN2: At baseline: S045: LATP; at follow-up: S047: TF-L, S046: RATP

Abbreviations: %: percentage, HIV: human immunodeficiency virus, LATP: left anterior tonsillar pillar, OR: oral rinse, PLWH: people living with HIV, RATP: right anterior tonsillar pillar, SN: seronegative, TF-L: tonsillar fossa-left, TF-R: tonsillar fossa-right

**Supplementary Figure 12:** **The mean relative abundance of the top 20 genera faceted by each subject at baseline and at follow-up (post-tonsillectomy)**

**Legend:** The mean relative abundance of the top 20 genera is shown across all 3 anatomical sites and stratified by HIV status. The heatmap was generated using ampvis2 in R.

Abbreviations: %: percentage, HIV: human immunodeficiency virus, PLWH: people living with HIV, SN: seronegative

**Supplementary Figure 13: Alpha diversity (A. Richness, B. Shannon) at baseline and at follow-up (after tonsillectomy)**


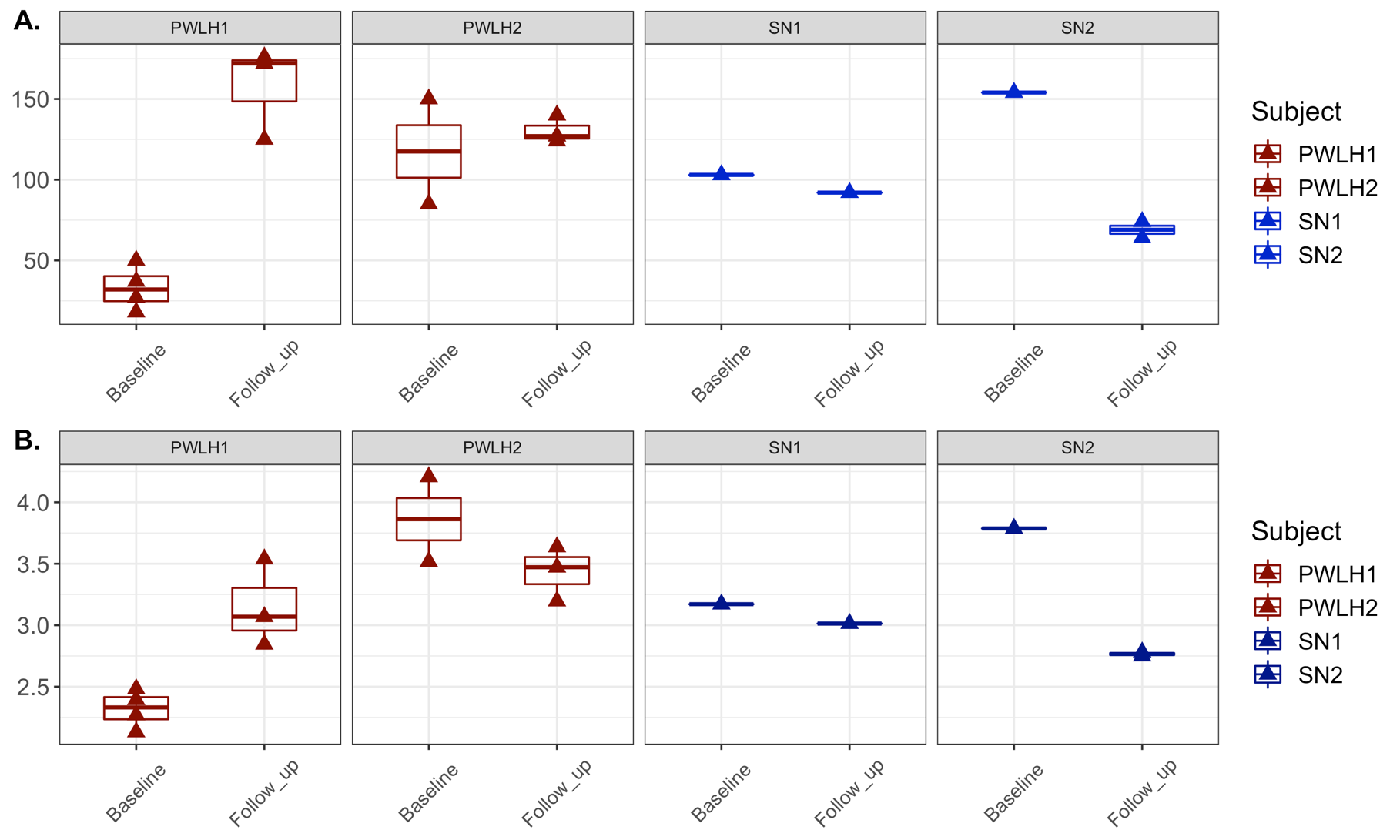


**Legend:** Two alpha metric were calculated: A. Richness (observed species) and B. shannon at baseline and at follow-up. Paired statistical analysis was not possible due to the lack of available paired samples at baseline and follow-up.

Abbreviations: HIV: human immunodeficiency virus, PLWH: people living with HIV, SN: seronegative

**Supplementary Figure 14: Beta diversity of microbial communities at baseline and at follow-up (after tonsillectomy)**


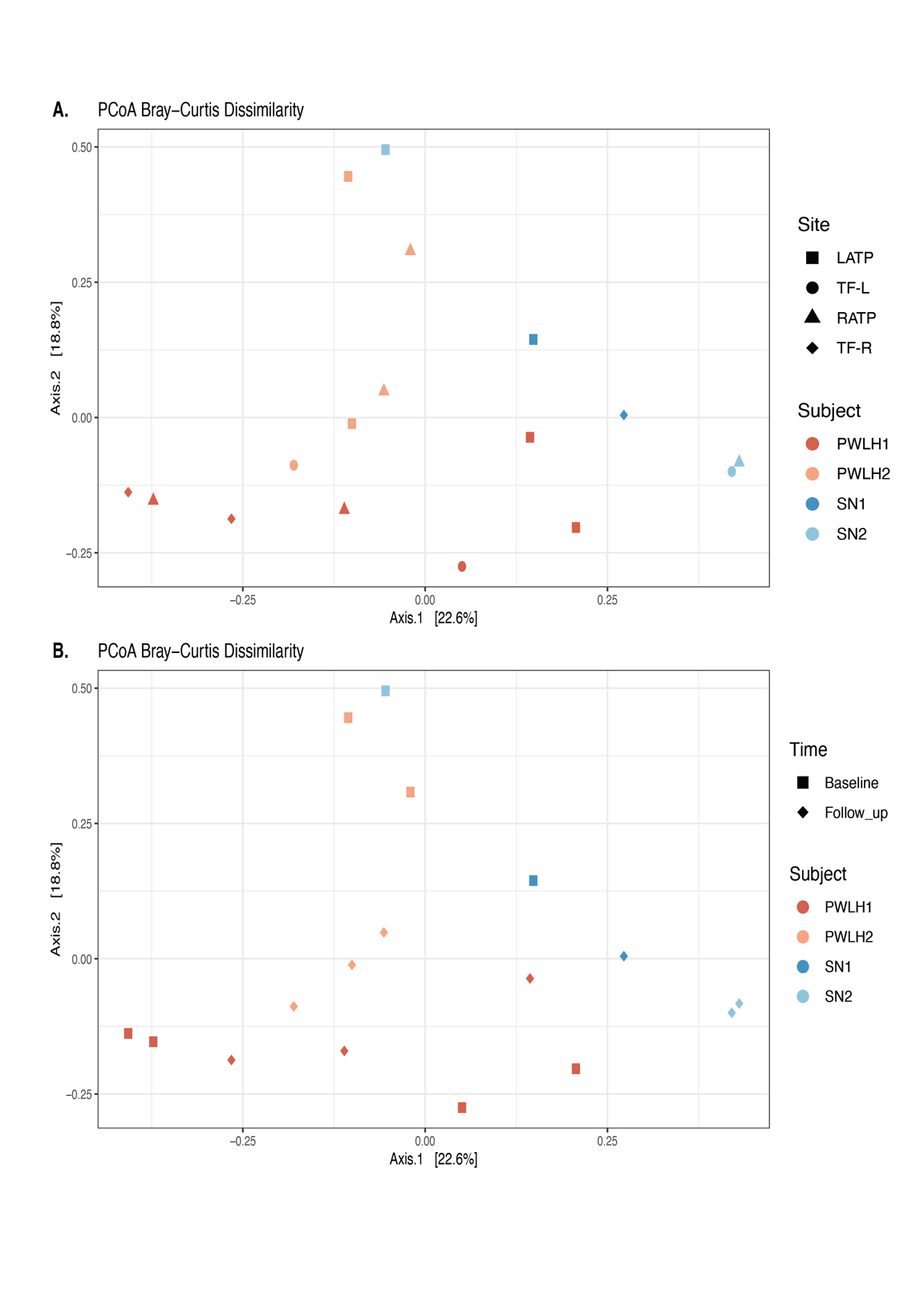


**Legend:** Clustering of microbial communities was visualized A. by anatomical site, B. by Time, using principal coordinate analysis (PCoA, Bray-Curtis dissimilarity) and differences were assessed by PERMANOVA after testing for homogeneity of dispersions (betadisper). Both subject (R2= 0.37, PERMANOVA p=0.001) and time (R2= 0.10, PERMANOVA p=0.009) impacted the oropharyngeal microbiota.

Abbreviations: HIV: human immunodeficiency virus, LATP: left anterior tonsillar pillar, PERMANOVA: Permutational multivariate analysis of variance, PLWH: people living with HIV, R2: R squared, SN: seronegative, RATP: right anterior tonsillar pillar, TF-L: tonsillar fossa-left, TF-R: tonsillar fossa-right

**Supplementary Figure 15: Alpha diversity by anatomical sites (nasopharynx, oropharynx and oral cavity) considering the overall cohort (including all subjects irrespective of their HIV status), PLWH and SN separately**

**Legend:** Differences in the bacterial microbiota was assessed by anatomical sites (NP: nasopharynx, OP: oropharynx and OR: oral cavity) considering the overall cohort (including all subjects irrespective of their HIV status), and PLWH and SN separately. Richness (number of observed species) was compared across anatomical sites, stratifying by HIV status and overall (including all subjects irrespective of their HIV status) using Kruskal-Wallis test, correcting all p values for multiple comparisons with Dunn´s multiple comparisons test.

*p<0.05, **p<0.01, ***p<0.001, ns: not significant

Abbreviations: HIV: human immunodeficiency virus, LATP: left anterior tonsillar pillar, NP: nasopharynx, OP: oropharynx, OR: oral rinse, PLWH: people living with HIV, RATP: right anterior tonsillar pillar, SN: seronegative, TF-L: tonsillar fossa-left, TF-R: tonsillar fossa-right

**Supplementary Figure 16. Differentially expressed genera were identified using LEfSe, with an LDA score of 4 or above.**

1. Overall (without considering HIV status)


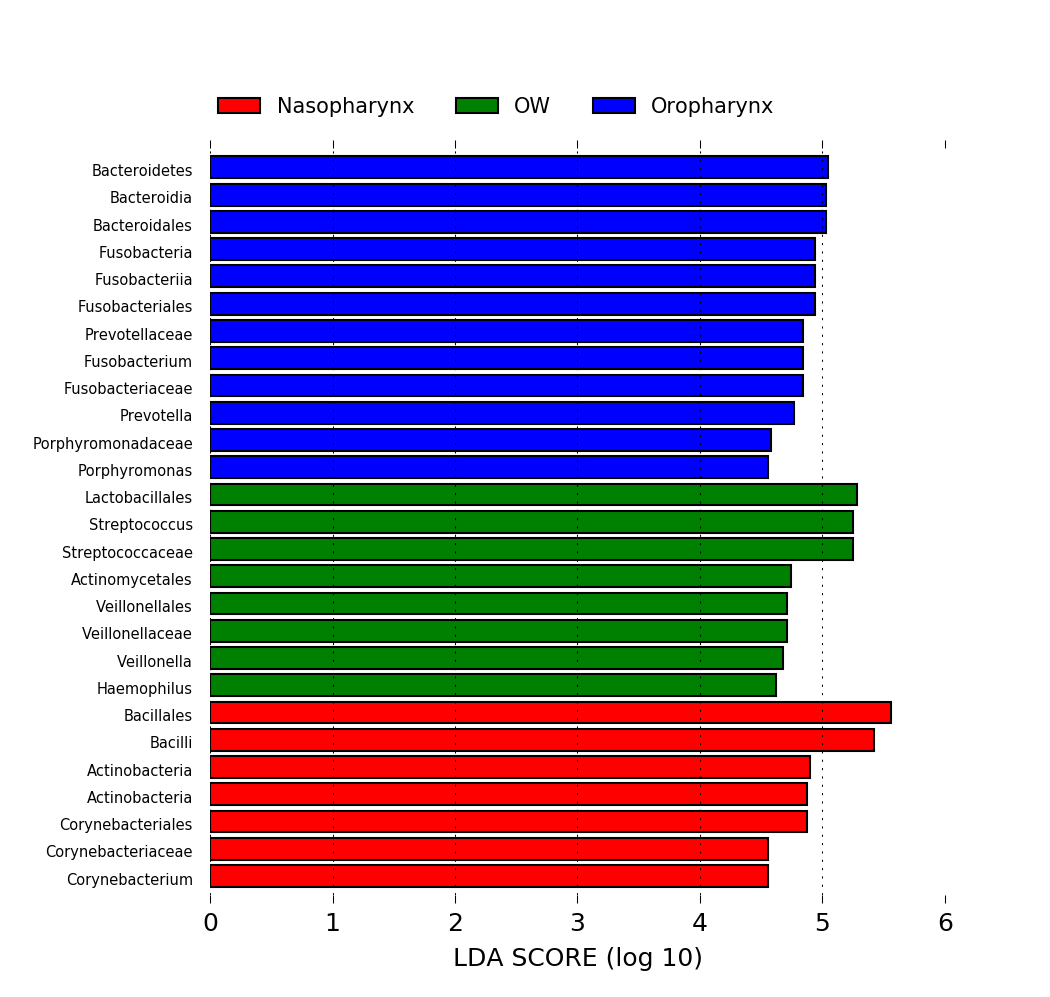


1. In Seronegative individuals


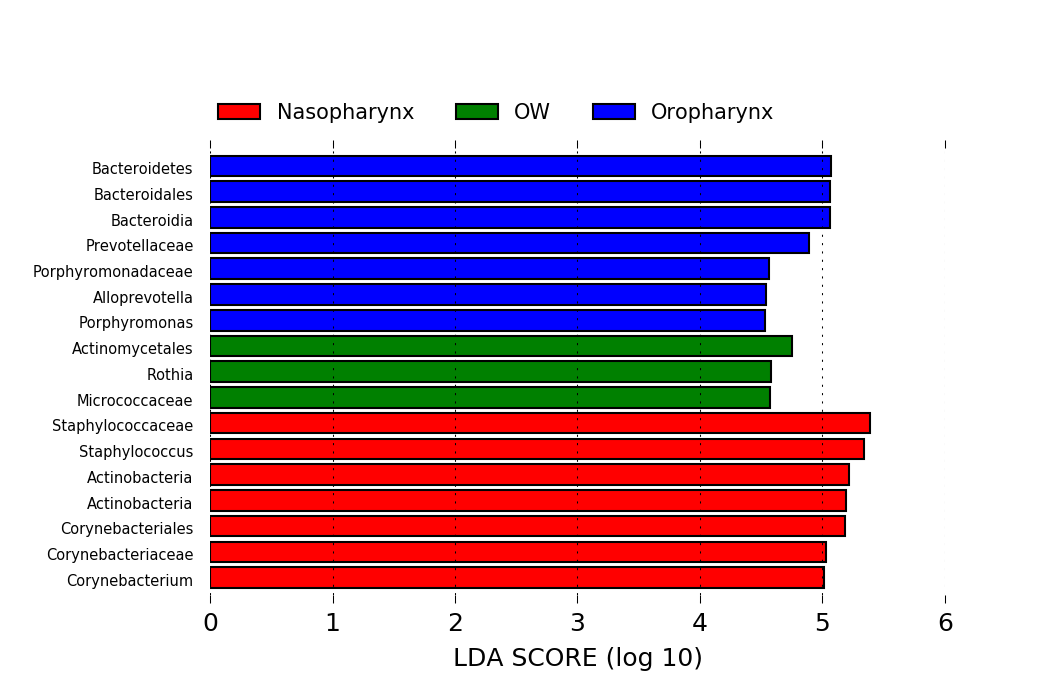


1. In People living with HIV

**
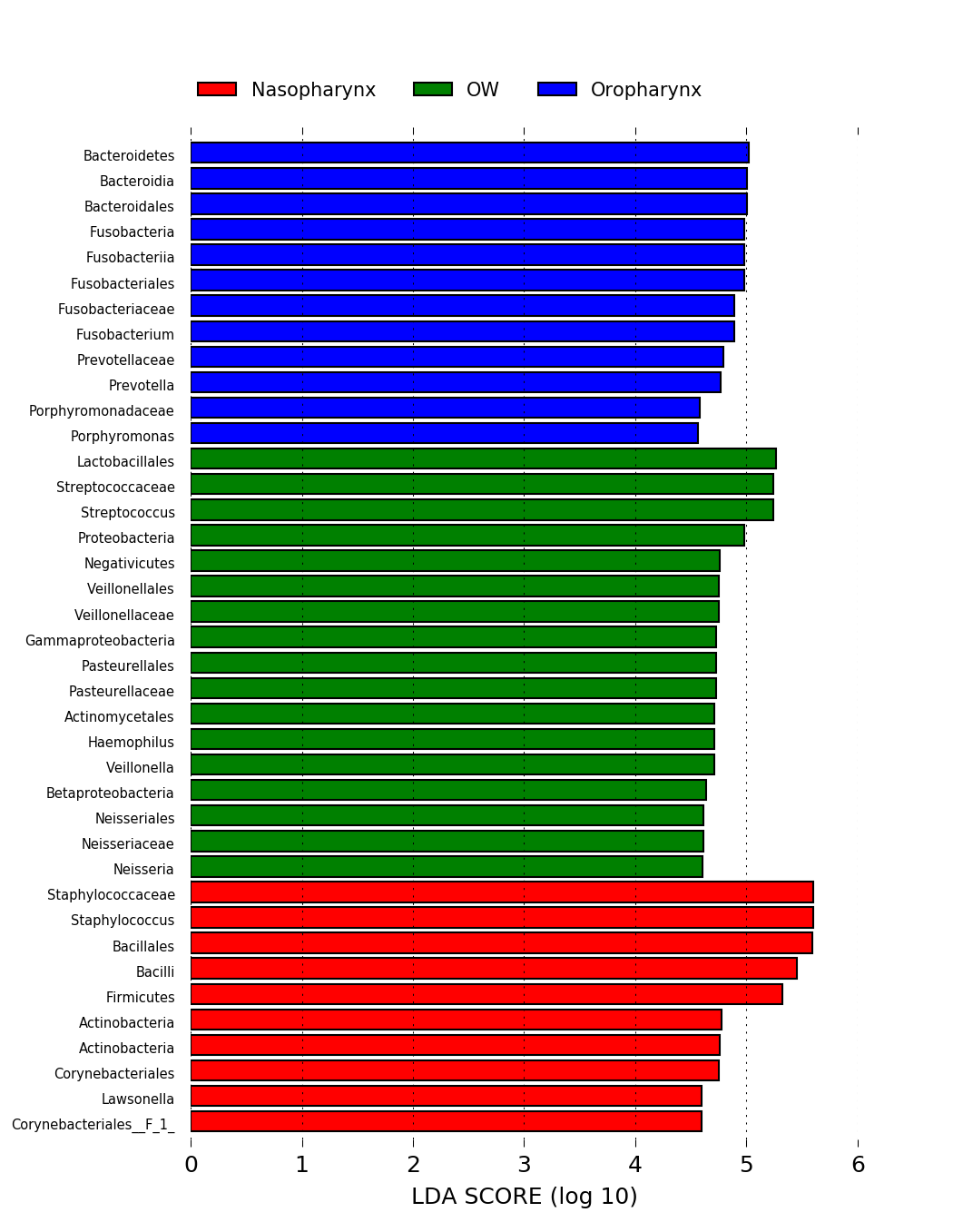
**

**Legend:** Differentially expressed genera were identified using LEfSe, with an LDA score of 4 or above.

Abbreviations: HIV: Human Immunodeficiency virus, LEfSe: Linear Discriminant Analysis effect size, NP: nasopharynx, OP: oropharynx, OW: oral wash/oral cavity, PLWH: People living with HIV, SN: Seronegative

**Supplementary Table 10: List of reagents, materials, instruments, and software used in this study**

| **Reagent/Resource/** **Instrument/Software** | **Identifier** | **Source** |
| --- | --- | --- |
| **Tissue collection and processing** | | |
| RPMI-1640 | Cat# 12633012 | Lonza, CA, USA |
| Fetal bovine serum (FBS) | Cat# 26140079 | Gibco, MA, USA |
| Penicillin/Streptomycin | Cat# 15140122 | Gibco, MA, USA |
| L-Glutamine | Cat# A2916801 | Gibco, MA, USA |
| Nylon mesh filter (70 µM) | Cat# 352350 | BD Biosciences, New Jersey, USA |
| CK (Chloride-Potassium) lysis buffer | Cat# 10-548E | Lonza, CA, USA |
| Phosphate-buffered saline (PBS) | Cat# 17-516F | Lonza, CA, USA |
| **Immunophenotyping of adenoids and tonsils** | | |
| eBiosciences FOXP3/fixation permeabilization kit | Cat# 00-5523-00 | ThermoFisher Scientific, MA, USA |
| Stain Buffer (FBS) | Cat# 554656 | BD Biosciences, New Jersey, USA |
| Paraformaldehyde | Cat# 158127 | Sigma-Aldrich, Misuri, USA |
| BD Cytometer Setup & Tracking Beads | Cat# 655050 | BD Biosciences, New Jersey, USA |
| BD Comp Beads | Cat# 552843, 552844 | BD Biosciences, New Jersey, USA |
| Corning Falcon Round-Bottom Polystyrene Test Tubes | Cat# 352054 | ThermoFisher Scientific, MA, USA |
| FACS Flow 20L | Cat**#** 342003 | BD Biosciences, New Jersey, USA |
| **Antibodies** | | |
| **Panel 1** | | |
| CD3/BV570 | Cat# 300436 Clone: UCHT1 | Biolegend, CA, USA |
| CD4/APC-Cy7 | Cat# 357416 Clone: A161A1 | Biolegend, CA, USA |
| RORγt/BV421 | Cat# 563282 Clone: Q21-559 | BD Biosciences, New Jersey, USA |
| CD38/BV711 | Cat# 303528 Clone: HIT2 | Biolegend, CA, USA |
| HLA-DR/BV785 | Cat# 307642 Clone: L243 | Biolegend, CA, USA |
| CD45RO/BV650 | Cat# 304232 Clone: UCHL1 | Biolegend, CA, USA |
| CRTH2/FITC | Cat# 350108 Clone: BM16 | Biolegend, CA, USA |
| CD25/PECy7 | Cat# 302612 Clone: BC96 | Biolegend, CA, USA |
| FOXP3/PE-CF594 | Cat# 320126 Clone: 206D | Biolegend, CA, USA |
| T-bet/PE | Cat# 561268 Clone: 04-16 | BD Biosciences, New Jersey, USA |
| CD161/APC | Cat# 339912 Clone: HP-3G10 | Biolegend, CA, USA |
| Live/dead/AmCyan | Cat# L34957 | ThermoFisher Scientific, MA, USA |
| CD8/BV605 | Cat# 339916 Clone: HP-3G10 | Biolegend, CA, USA |
| CD19/BV510 | Cat# 302242 Clone: HIB19 | Biolegend, CA, USA |
| CD14/BV510 | Cat# 301842 Clone: M5E2 | Biolegend, CA, USA |
| CD56/BV510 | Cat# 563041Clone: HCD56 | Biolegend, CA, USA |
| CD11c/BV510 | Cat# 301634 Clone: 3.9 | Biolegend, CA, USA |
| CD123/BV510 | Cat# 306022 Clone: 6H6 | Biolegend, CA, USA |
| **Panel 2** | | |
| CD3/BV570 | Cat# 300436 Clone: UCHT1 | Biolegend, CA, USA |
| CD4/PE | Cat# 357404 Clone: A161A1 | Biolegend, CA, USA |
| CD14/Pacific Blue | Cat# 301828 Clone: M5E2 | Biolegend, CA, USA |
| CD56/BV711 | Cat# 318336 Clone: HCD56 | Biolegend, CA, USA |
| CXCR5/PECF-594 | Cat# 356928 Clone: J252D4 | Biolegend, CA, USA |
| HLA-DR/BV785 | Cat# 307642 Clone: L243 | Biolegend, CA, USA |
| CD19/BV650 | Cat# 302238 Clone: HIB19 | Biolegend, CA, USA |
| PD-1/BV605 | Cat# 329924 Clone: EH12.2H7 | Biolegend, CA, USA |
| CD16/APCCy7 | Cat# 302018 Clone: 3G8 | Biolegend, CA, USA |
| Live/dead/AmCyan | Cat# L34957 | ThermoFisher Scientific, MA, USA |
| CD69/PECy7 | Cat# 310912 Clone: FN50 | Biolegend, CA, USA |
| CD123/APC | Cat# 560087 Clone: 9F5 | BD Biosciences, New Jersey, USA |
| CD11c/PECy5 | Cat# 551077 Clone: B-ly6 | BD Biosciences, New Jersey, USA |
| **Sample collection for microbial 16S sequencing** | | |
| Dacron Swab | Cat# 11582483 | ThermoFisher Scientific, MA, USA |
| Solución CS PiSA® |  | PiSA, Guadalajara, Mexico |
| 15 ml threaded tube, PP conical bottom (swabs) | Cat# 50015 | SPL Life Sciences, Gyeonggi, Korea |
| 50 ml threaded tube, PP conical bottom (OW) | Cat# 50050 | SPL Life Sciences, Gyeonggi, Korea |
| **DNA extraction and 16S rDNA sequencing** | | |
| PowerSoil® DNA Isolation Kit | Cat# 12888-100 | MO BIO, CA, USA |
| Ethanol | Cat# E7023  Lot# SHBF2298V | Sigma-Aldrich, Misuri, USA |
| Eppendorf DNA LoBind Polypropylene Tubes (1.5 mL) | Cat# 10051232  Lot# D157243M | Eppendorf, MA, USA |
| Amplicon PCR Reverse Primer V3V4 (16s V3V4R- 5’-  GACTACHVGGGTATCTAATCC-3´: 10nM) | Cat# 10336022 | Invitrogen, MA, USA |
| Amplicon PCR Forward Primer V3V4 (16s V3V4F- 5’-CCTACGGGNGGCWGCAG-3´: 10nM) | Cat# 10336022 | Invitrogen, MA, USA |
| MicroAmp Optical 96-well Reaction Plate | Cat# N8010560 | ThermoFisher Scientific, MA, USA |
| Dimethyl Sulfoxide for molecular biology (DMSO) | Cat# D8418  Lot# 02696BM | Sigma-Aldrich, Missouri, USA |
| Platinum *Taq* DNA Polymerase High Fidelity 100 rxn:  Platinum *Taq* DNA High Fidelity Polymerase 5U/µL (20 µL)  10x High Fidelity PCR Buffer (600 mM Tris-SO4, (pH 8.9), 180 mM (NH4)2SO4 (1.25 mL)  50 nM MgSO_4_ (1 mL) | Cat# 11304-011  Lot#1880837 | Invitrogen, MA, USA |
| 10nM dNTP Mix | Cat# 18427-013  Lot# 1876283 | Invitrogen, MA, USA |
| Certified Molecular Biology Agarose, 500g | Cat# 1613102 | BIO-RAD, CA, USA |
| Agencourt AMPure XP beads-PCR Purification | Cat# A63880  Lot# 16585900 | Beckman Coulter, CA, USA |
| Magnetic Stand-96 | Cat# AM10027  Lot# 00287506 | Invitrogen, MA, USA |
| Nextera XT Index Kit v2 Set A | Cat# 15052163  Lot# 20135514 | Illumina, CA, USA |
| Qubit assay tubes  Qubit dsDNA HS Buffer and Standards | Cat# Q32856  Cat# Q32851 | Invitrogen, MA, USA |
| Agilent High Sensitivity DNA Kit | Cat# 5067-4626 | Agilent Technologies, CA, USA |
| NaOH | Cat# 1310-73-2 | Sigma-Aldrich, Misuri, USA |
| MiSeq Reagent Kits v3 (600 Cycles) | Cat# 15043895, 15043894  Lot# 20103416, 20115812 | Illumina, CA, USA |
| Tween 20 | Cat# P9416-100  Lot# SLBP6514V | Sigma-Aldrich, Misuri, USA |
| PhiX Control Kit v3 | Cat# FC‐110‐3001 | Illumina, CA, USA |
| **Critical commercial assays** | | |
| PowerSoil® DNA Isolation Kit | Cat# 12888-100 | MO BIO, CA, USA |
| 16S Metagenomic Sequencing Library  Preparation | Cat# 15044223 | Illumina, CA, USA |
| LIVE/DEAD Fixable Aqua Dead Cell  Stain Kit | Cat# L34957 | ThermoFisher Scientific, MA, USA |
| **Instrument** | | |
| Fortessa LSR cytometer |  | BD Biosciences, New Jersey, USA |
| Sorvall Legend Micro 17 centrifuge, ventilated | Cat# 75002430 | ThermoFisher Scientific, MA, USA |
| Thermo NanoDrop 1000 Spectrophotometer | Cat# E112352 | ThermoFisher Scientific, MA, USA |
| MO BIO Vortex Adapter (24 tubes) | Cat# 13000-V1-24 | MO BIO, CA, USA |
| Vortex-Genie 2 | Cat# 50-728-002 | Scientific Industries, New York, USA |
| Veriti® 96-Well Thermal Cycler | Cat# 4375786 | ThermoFisher Scientific, MA, USA |
| Qubit 3.0 Fluorometer | Cat# Q33216 | Invitrogen, MA, USA |
| 2100 Bioanalyzer Instrument | Cat# G2939BA | Agilent Technologies, CA, USA |
| MiSeq, instrument | Cat# SY-410-1003 | Illumina, CA, USA |
| **Softwares** | | |
| FlowJo software | Version 10 | https://www.flowjo.com |
| Illumina MiSeq platform | NA | https://www.illumina.com |
| QIIME2 | Version 2019.4 | https://qiime2.org |
| R libraries | Version 3.6.2 | https://www.r-project.org |
| Linear discriminant analysis (LDA)  effect size (LEfSe) | NA | https://huttenhower.sph.harvard.edu/lefse |
| GraphPad Prism | Version 9 | https://www.graphpad.com |

Abbreviations: APC: Allophycocyanin, BD: Becton Dickinson, BV: Brilliant Violet, Cat: Catalogue, CD: cluster of differentiation, CK: Chloride-Potassium, CRTH2: chemoattractant receptor-homologous molecule expressed on TH2 cells, DMSO: Dimethylsulfoxide, DNA: Deoxyribonucleic acid, dNTP: deoxynucleoside triphosphate, F: forward, FBS: Fetal bovine serum, FITC: Fluorescein isothiocyanate, FOXP3: forkhead box P3, HLA-DR: human leucocyte antigen-DR, LEfSe: Linear discriminant analysis effect size, LDA: Linear discriminant analysis, MgSO4: magnesium sulfate, μL: microliter, mL: milliliter, µM: micromolar, mM: millimolar, NaOH: Sodium hydroxide, (NH4)2SO4: ammonium sulfate, PBS: Phosphate-buffered saline, PCR: polymerase chain reaction, PE: Phytoerythrin, PP: polypropylene, QIIME2: Quantitative Insights Into Microbial Ecology 2, R: reverse, RORγt: retinoic acid-related orphan receptor-gamma, RPMI: Roswell Park Memorial Institute medium, Tris-SO4: trisaminomethane sulfate, U: units.
